# Lack of a Cytoplasmic RLK, Required for ROS Homeostasis, Induces Strong Resistance to Bacterial Leaf Blight in Rice

**DOI:** 10.3389/fpls.2018.00577

**Published:** 2018-05-18

**Authors:** Youngchul Yoo, Jong-Chan Park, Man-Ho Cho, Jungil Yang, Chi-Yeol Kim, Ki-Hong Jung, Jong-Seong Jeon, Gynheung An, Sang-Won Lee

**Affiliations:** ^1^Graduate School of Biotechnology and Crop Biotech Institute, Kyung Hee University, Yongin, South Korea; ^2^Institute for Molecular Physiology, Heinrich-Heine-Universität Düsseldorf, Düsseldorf, Germany; ^3^Max Planck Institute for Plant Breeding Research, Cologne, Germany

**Keywords:** peroxisomal biogenesis factor 11, vascular one zinc-finger 1, plant defense, reactive oxygen species, ROS-homeostasis, *Xanthomonas oryzae* pv. *oryzae*

## Abstract

Many scientific findings have been reported on the beneficial function of reactive oxygen species (ROS) in various cellular processes, showing that they are not just toxic byproducts. The double-edged role of ROS shows the importance of the regulation of ROS level. We report a gene, *rrsRLK* (required for ROS-scavenging receptor-like kinase), that encodes a cytoplasmic RLK belonging to the non-RD kinase family. The gene was identified by screening rice RLK mutant lines infected with *Xanthomonas oryzae* pv. *oryzae* (*Xoo*), an agent of bacterial leaf blight of rice. The mutant (Δ*rrsRLK*) lacking the *Os01g02290* gene was strongly resistant to many *Xoo* strains, but not to the fungal pathogen *Magnaporthe grisea*. Δ*rrsRLK* showed significantly higher expression of *OsPR1a*, *OsPR1b*, *OsLOX*, *RBBTI4*, and jasmonic acid-related genes than wild type. We showed that rrsRLK protein interacts with OsVOZ1 (vascular one zinc-finger 1) and OsPEX11 (peroxisomal biogenesis factor 11). In the further experiments, abnormal biogenesis of peroxisomes, hydrogen peroxide (H_2_O_2_) accumulation, and reduction of activity of ROS-scavenging enzymes were investigated in Δ*rrsRLK*. These results suggest that the enhanced resistance in Δ*rrsRLK* is due to H_2_O_2_ accumulation caused by irregular ROS-scavenging mechanism, and rrsRLK is most likely a key regulator required for ROS homeostasis in rice.

## Introduction

Despite the massive efforts of international organizations and scientists to solve food problems, 108 million people world-wide face serious food insecurities (Arthur, 2008). Furthermore, the growth rate of the global population demands continued efforts to supply more food to humans. There are diverse causes that reduce the productivity of crops, and losses by biotic invaders are serious. These situations illustrate the importance of research on crop defense systems against small pathogens such as viruses, bacteria, and fungi.

Since the gene-for-gene hypothesis was proposed (Flor, 1971), plant defense systems have been largely concentrated in two types. Both are known to be triggered by interactions between pathogen elicitors and host plant receptors. The first one is rapidly triggered by interactions between pathogen-associated molecular patterns (PAMPs, e.g., chitin, flagellin, and elongation factor-Tu) from pathogens and pattern recognition receptors (PRRs, e.g., chitin elicitor receptor kinase 1, flagella sensing 2, elongation factor-Tu receptor, and Rpg1) of host plants (Brueggeman et al., 2002; Kunze et al., 2004; Chinchilla et al., 2006; Miya et al., 2007). The defense system is called PAMP-Triggered Immunity (PTI), and the interactions mostly occur extracellularly. PAMP-PRR interactions typically support the activation of mitogen-activated protein kinases, the rapid production of reactive oxygen species (ROS), and the induction of pathogenesis-related (*PR*) gene expression (Pitzschke et al., 2006). Plants also have cytoplasmic receptors that sense pathogenic effectors delivered through type three secretion systems (Jones and Dangl, 2006). We call the effectors and receptors avirulence (Avr) factors and disease resistance (R) proteins, respectively. The second type of defense system (called Effector-Triggered Immunity, ETI) usually causes programmed cell death (Cunnac et al., 2009) and induces much stronger and strain-specific responses compared to PTI (Jones and Dangl, 2006). ETI is often triggered indirectly. R protein senses interaction of pathogen effector and host protein (called a guardee) and triggers ETI (Block et al., 2008). In response to the defense mechanisms of host plants, pathogens have evolved to avoid recognition by receptors for PTI/ETI through either lost or altered effector (Thomma et al., 2011), but host plants have also evolved to detect such changes in pathogens (Almagro et al., 2009).

Although the pathogen molecules recognized by the plant receptors in ETI or PTI are diverse and their interactions with plant receptors are very specific, receptors responsive to PTI or ETI show high similarity in protein structure. The majority of R proteins in ETI typically possess a nucleotide binding site-leucine rich repeat harboring a variable N-terminus such as coiled-coil or toll/interleukin 1 receptor like domains (Boller and Felix, 2009). PRRs typically contain a receptor domain, frequently followed by a protein kinase (Braun and Walker, 1996). Alternatively, the receptor has a protein kinase as a signaling partner (Medzhitov, 2001). Concerning PRRs, members of the interleukin-1 receptor-associated kinase (IRAK) family involved in immune responses of animals and plants are distinguishable from members unrelated to defense (Dardick and Ronald, 2006). Dardick and Ronald (2006) suggested that most known or predicted PRRs have a kinase domain that belongs to a non-arginine (R)-aspartate (D) kinase group. It is well-known that conserved R residue immediately preceding the conserved catalytic D residue in the catalytic loop is critical for phosphorylation processes in a group of kinases (so called RD kinases) (Nolen et al., 2004). However, most kinases associated with PTI replace the positively charged R in the catalytic loop with uncharged residues such as Cys, Gly, Phe, or Leu with an exception of *Arabidopsis* chitin elicitor receptor kinase (CERK1) (Miya et al., 2007). In the rice genome, 371 of over 1,000 IRAKs (Zhou et al., 2004) have the non-RD motif (Dardick and Ronald, 2006), although only a small number of these have been functionally characterized.

We carried out inoculation tests with an *IRAK* gene mutant rice population (suppression or activation) carrying the non-RD motif using a pathogenic bacterium, *Xanthomonas oryzae* pv. *oryzae* (*Xoo*). *Xoo*, an agent of leaf blight in rice, is a major contributor to serious reduction of rice production in Asia and Africa (Khan et al., 2014). In the test, we selected genes that are most likely involved in rice defense. We report an interesting gene in this study, named *rrsRLK* (required for ROS-scavenging receptor-like kinase), that regulates ROS levels in rice and plays a negative role in defense against *Xoo* infection.

## Materials and Methods

### Plants, Bacteria, and Fungi and Growth Conditions

An *IRAK* gene mutant rice population (generated with *Oryza sativa* var. japonica cv. Dongjin or Hwayoung) (Jeon et al., 2000) carrying the non-RD motif was used for inoculation tests with *Xoo* strains and resulted in selection of 3A-10392 (a knock-out line of Os01g02290 in Dongjin, Δ*rrsRLK*_dj_), 4A-01523 (an activation line of Os01g02290 in Dongjin, *rrsRLK_Act1_*), 2A-50012 (another activation line of Os01g02290 in Dongjin, *rrsRLK_Act2_*), and 3B-00367 (a knock-out line of Os01g02290 in Hwayoung, Δ*rrsRLK*_hy_) mutant rice plants. Additional knock-out mutant lines that carry a T-DNA insertion at *Os01g54930* encoding OsVOZ1 (rice vascular one zinc finger protein 1, K-05631) in Kittake (*O. sativa* var. japonica) and at *Os03g02590* encoding OsPEX11 (rice peroxisomal biogenesis factor 11, 1B-07040) in Dongjin were selected and used. All mutant lines, including wild types (Dongjin and Hwayoung) as controls, are listed in Supplementary Table S1, and the genotyping method to determine segregation family is described in the Supplementary Materials.

Seeds were germinated on petri dishes containing water-drenched filter paper at 28°C for three days, transferred to soil, and then grown in a green house or paddy field before pathogen inoculation. All the inoculation experiments were carried out with 6-week-old rice or 7-week-old rice plants (in the winter season). Inoculation tests were carried out in a restricted chamber with conditions of 28°C with 85% humidity for 14 h during the day and 25°C and 80% humidity for 10 h at night.

*Xoo* strains (PXO99A, Philippine strain 6, compatible with Dongjin, PXO99 in this study; HB01009, Korean strain 3a, compatible to Hwayoung) and *Magnaporthe grisea* (*M. grisea*, *PO6-6*, a Philippine isolate) strains used in this study are listed in Supplementary Table S1. *Xoo* strains were cultured at 28°C for three days on plates of peptone sucrose agar (PSA) medium (10 g/L of peptone, 10 g/L of sucrose, 1 g/L of glutamic acid, 16 g/L agar, pH 7.0) and used for inoculation tests. *M. grisea*, *PO6-6* was inoculated on potato dextrose (PDA) medium (24 g/L of potato starch, dextrose, 15 g/L agar) and cultured at 28°C for three days.

### Pathogen Inoculation and Disease Evaluation

Inoculation of *Xoo* strains and *M. grisea* was carried out using the clipping method (Kauffman et al., 1973) and the leaf punch inoculation method (Lee et al., 2009), respectively. All the details including pathogen preparation for inoculation and scoring lesion lengths on rice leaves are described in the Supplementary Materials.

### RT-PCR and Quantitative RT-PCR

RT-PCR and qRT-PCR were carried out to investigate expression of target genes such as *Os01g02290*, *Os01g54930, Os03g02590*, and *PR* genes in each mutant line. Total RNA extraction and cDNA synthesis were carried out using the method provided by the respective company, Takara (Japan) and Clontech Laboratories (United States). RT- and qRT-PCR analyses were repeated more than three times with three biological replicates. Thermo cycling conditions for RT- and qRT-PCR reactions are described in the Supplementary Materials, and used primers are listed in Supplementary Tables S2–S4.

### Sub-Cellular Localization of rrsRLK Protein

Full-length cDNA of *Os01g02290.2* was amplified by PCR using a cDNA clone (accession number: AK241355, KOME number: J065150L24) supplied from KOME^1^ and specific primers (Supplementary Table S3) containing recognition sequences of *Sma*I and *Spe*I restriction enzymes. The PCR product was inserted into the pGA3452 vector (Kim S.R. et al., 2009) after cutting with *Sma*I and *Spe*I. The pGA3452 vector is designed for expression of GFP fusion proteins under control of a maize ubiquitin promoter. The construct was then introduced into protoplast mesophyll and OC cells using the PEG transformation method (Choi et al., 2014) for transient expression of the *rrsRLK*-*sGFP* gene. Additional constructs, previously generated to express plasma membrane (PM)-mRFP (Kim et al., 2009) and nuclear localization signal (NLS)-mRFP (Kim et al., 2009) driven by 35S promoters, were also introduced into the protoplasts as markers. Signals of rrsRLK-sGFP, PM-mRFP, and NLS-mRFP were then examined on a fluorescence microscope Axioplan 2 (LSM 510 META; Zeiss, Germany) equipped with filter sets for GFP (excitation wavelength/dichroic transition: 488/543 nm) and RFP (excitation wavelength/dichroic transition: 561/575 nm). Methods for protoplast isolation (Yang et al., 2013) and transformation (Hong et al., 2012) were used for this study with minor modifications (see the Supplementary Materials).

### Yeast Two-Hybrid Assay

A Gal4-based system with Gateway technology (Invitrogen, United States) was used for a yeast two-hybrid assay. DNA fragment (nt 859–1197, *rrsRLK_k_*) corresponding to the kinase domain of rrsRLK protein was amplified by PCR with specific primers containing *attB*1 and *attB*2 sites and KOME full-length cDNA of *Os01g02290.2* as a template. The PCR product was cloned into pDONR222 (Invitrogen, United States) by BP recombination to generate the entry clone. Afterward, *rrsRLK_k_* was transferred to the yeast destination bait plasmid pDEST32 (Invitrogen, United States) by LR recombination, resulting in pDEST32rrsRLK_k_ (pD32rrsRLK_k_). To construct a Dongjin cDNA library, cDNA of approximately 0.5–3 kbp was cloned into pDONR222 and subsequently into pDEST22 by LR recombination. This yielded pD22Lib (pDEST22 containing the 0.5–3 kb fragment of rice cDNA Library, Amp^R^). pD32rrsRLK_k_ contains the DNA-binding domain of Gal4 and the leucine selection marker gene LEU2. pD22Lib contains the GAL4 transcription activation domain and the tryptophan selection marker gene *TRP1*. All constructs were checked by restriction enzyme analysis and confirmed by DNA sequencing. pD32rrsRLK_k_ and pD22Lib were co-transformed into yeast strain YD116 cells according to the manufacturer’s protocol (Invitrogen, United States). The transformants were cultured on synthetic complete medium, lacking leucine (-Leu) and tryptophan (-Trp). After 72 h, the colonies were picked and mixed with 100 μL of sterile water. Then, 10 μL of the cell suspension was spotted onto selection plates to screen for expression of the three reporter genes (*HIS3*, *URA3*, and *lacZ*). Growth of the yeast was assessed on SC-Leu-Trp-His supplemented with SC-Leu-Trp-Ura and 0-50 mM 3-amino-1, 2, 4-triazole (3AT) as a histidine inhibitor. A change in the blue color of the transformants was monitored for the presence of X-Gal (5-bromo-4-chloro-3-indolyl-β-d-galactopyranoside). pD32rrsRLK_k_, pD22OsVOZ1, and pD22OsPEX11 were combined with pDEST32 or pDEST22 and tested for auto-activation of the reporter genes.

### *In Vivo* Coimmunoprecipitation Assays

A coimmunoprecipitation assay was carried out using the previously reported method with minor modifications (Yang et al., 2013). Constructs were generated for transient expression of HA-tagged rrsRLK, Myc-tagged OsVOZ1, and Myc-tagged PEX11. Full-length *rrsRLK* without the stop codon was amplified by PCR using the primer sets listed in Supplementary Table S2 and inserted into the *Hpa*I/*Kpn*I sites of the HA-tagged vector pGA3698. The pGA3698 vector contains the maize Ubiquitin1 promoter and the 3x HA coding region. pGA3697 vectors carrying full-length *OsVOZ1* and *OsPEX11* were constructed using *Hpa*I. The pGA3697 vector carried the maize Ubiquitin1 promoter and the 4x Myc coding region. To verify the interaction of rrsRLK-OsVOZ1 and rrsRLK-OsPEX11, a combination of the constructs was transformed into the protoplasts isolated from rice mesophyll or OC cells using PEG-mediated transformation (see Supplementary Materials). The protoplasts were re-suspended in IP buffer [50 mM Tris–HCl, pH 7.5, 1 mM EDTA, 150 mM NaCl, 1% (v/v) Triton X-100, 1 mM DTT, 2 mM NaF, 50 μM MG132, and an adequate amount of protease inhibitor cocktail (Roche, United States)]. After vortexing briefly, the samples were centrifuged at 12,000 rpm for 10 min. Next, 10 μL each of protein A and G conjugated to agarose beads (Millipore, United States) were added to the supernatant for pre-clearing to prevent non-specific binding. We used 10% of the pre-cleared extracts as input controls, while the rest was precipitated with an anti-HA monoclonal antibody (Roche, United States) and incubated overnight with gentle shaking. The incubated sample was then mixed with 10 μL each of protein A and G conjugated to agarose beads and incubated for 2 h. After being washed five times with IP buffer, the proteins bound to the beads were eluted with 20 μL of SDS sample buffer. The eluents were separated with electrophoresis on a 10% SDS-polyacrylamide gel and transferred to a PVDF membrane (Millipore, United States). The experiment for monitoring the interaction between rrsRLK, OsVOZ1, and OsPEX11 was performed multiple times using the anti-Myc monoclonal antibody for precipitation and an HRP-conjugated anti-HA monoclonal antibody (Cell Signaling Technology, United States) for detection. Proteins that bound to the membrane were incubated with an HRP-conjugated anti-Myc monoclonal antibody (Cell Signaling technology, United States) and detected using an ECL prime western blotting detection reagent (GE Healthcare, United Kingdom) in LAS-4000 (GE Healthcare, United Kingdom).

### H_2_O_2_ Measurement by Enzymatic Assay

The roots, stems, and leaves of *Se-WT_dj_* and Δ*rrsRLK*_dj_ (1g each) were homogenized in 100 mM sodium phosphate buffer (pH 6.8). The homogenate was filtered through four layers of gauze and centrifuged at 17,000 × *g* for 25 min at 4°C. The supernatant was collected for measurement of H_2_O_2_ contents. Contents of H_2_O_2_ in rice tissues were determined by a modified method of Bernt and Bergmeyer (1974) using peroxidase. To initiate the enzyme reaction, an aliquot of 0.5 mL of supernatant was mixed with 2.5 mL of peroxide reagent consisting of 83 mM sodium phosphate (pH 7.0), 0.005% (w/v) o-dianisidine, and 40 μg/mL of peroxidase (Sigma-Aldrich, United States). After 15 min incubation at room temperature, the reactions were stopped by adding 0.5 mL of 1 N perchloric acid and centrifuged at 5,000 × *g* for 5 min. The absorbance of the sample was measured at 436 nm and was compared to the extinction of a H_2_O_2_ standard for quantitative analysis.

### Analysis of Antioxidant Enzyme Activity in Δ*rrsRLK*_dj_

To extract total protein from leaves of WT and Δ*rrsRLK*_dj_, 1 g leaves was homogenized in 100 mM potassium phosphate buffer (pH 7.8) containing 0.1 mM EDTA, 1% (w/v) PVP (Polyvinylpyrrolidone), and 0.5% (v/v) Triton X-100 at 4°C. The homogenate was filtered through four layers of cheesecloth and centrifuged at 17,000 × *g* for 25 min at 4°C. The supernatant was collected for measurement of the enzymatic activities of superoxide dismutase (SOD), ascorbate peroxidase (APX), catalase (CAT), and peroxidase (POX). SOD activity was determined based on the method described by Beyer and Fridovich (1987) with a minor modification. The reaction mixture (1.06 mL) was composed of 50 mM potassium phosphate (pH 7.8), 9.9 mM methionine, 57 μM nitro blue tetrazolium, 0.025% triton X-100, 0.4 μM riboflavin, and the 40 μL extract. The mixture was incubated with irradiation of light for 7 min, and absorbance was monitored at A_560__nm_. To measure APX activity, the protein extract was added to reaction mixture containing 0.5 mM ascorbate and 0.2 mM H_2_O_2_ in 990 μL of 50 mM phosphate buffer (pH 7.0), and absorbance was monitored at 290 nm for 10 min. CAT activity was determined spectrophotometrically. Protein extract (1.92 mL) was mixed with 80 μL of 50 mM phosphate buffer (pH 7.0) containing 30 mM H_2_O_2_ as a substrate, and absorbance at 240 nm was monitored for 10 min. POX activity was determined by monitoring the formation of guaiacol dehydrogenation product (extinction coefficient 6.39 mM/L cm^-1^) at 436 nm. Reaction mixture contained 100 mM potassium phosphate (pH 7.0), 0.3 mM guaiacol, and 50 μL of the protein extract. The reaction was initiated by adding 0.1 mM H_2_O_2_, and A_436__nm_ was monitored for 10 min.

## Results

### Lack of the *Os01g02290* Gene Induces Strong Resistance Against *Xoo*

**FIGURE 1:**
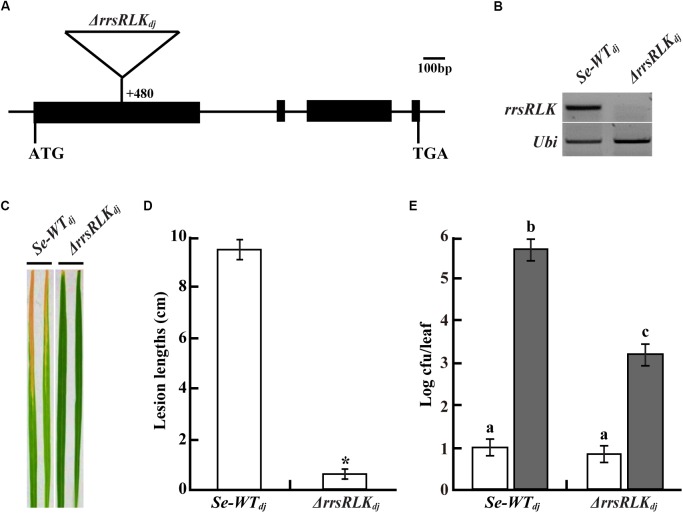
**(A)** Schematic representation of T-DNA insertion (+480 bp from the translational start site) at *Os01g02290* encoding rrsRLK in Dongjin (Δ*rrsRLK*_dj_). ATG start codon and TGA stop codon are indicated. *Os01g02290* consists of four exons (black boxes) and three introns (line between the black boxes). **(B)** Expression analysis of the *rrsRLK* gene in *SE-WT_dj_* (segregated wild type) and Δ*rrsRLK*_dj_ mutant lines using RT-PCR with *rrsRLK* primers. *Ubi* gene was used as an internal control. The experiment was repeated three times with consistent results. **(C)** Comparison of lesion development on leaves of *SE-WT_dj_* and homozygous mutant lines of Δ*rrsRLK*_dj_. Rice plants grown for 6 weeks were inoculated with PXO99 and monitored for lesion development for 14 DAI. The results were consistent until the 6th generation of the mutant, and each experiment was carried out with more than 40 leaves. **(D)** Lesion lengths scored at 14 DAI on leaves of *SE-WT_dj_* and Δ*rrsRLK*_dj_ mutant lines. The averages and error ranges of each sample were calculated from 40 leaves. Asterisk indicates *P* < 0.05 (Student’s *t*-test). Data in **(C,D)** are results of experiments carried out using the 3rd generation of Δ*rrsRLK*_dj_ mutant lines. **(E)** Bacterial population extracted from inoculated leaves of *SE-WT_dj_* and Δ*rrsRLK*_dj_ mutant lines at 0 and 14 DAI. *Xoo* was extracted from inoculated leaves and incubated on PSA plates after serial dilution for colony counting. Each data point represents the average and standard deviation of three biological replicates. The experiment was repeated three times with similar results. Different letters above bars indicate statistically significant differences as determined by one-way analysis of variance (ANOVA: ^∗^, a, b, c), *P* < 0.05.

We selected 68 mutant lines from a T-DNA rice mutant population (Jeon et al., 2000). The mutant lines have T-DNA insertion into *IRAK* genes carrying a non-RD kinase, resulting in suppression or activation of the gene. Inoculation tests using the mutant lines and PXO99 resulted in the selection of a few mutant lines showing shorter lesion lengths than Dongjin rice. One of these is a mutant line (3A-10392, named Δ*rrsRLK*_dj_ in this study) where *Os01g02290* gene expression was suppressed by T-DNA insertion into the first exon (nt 480) (**Figures 1A,B**). Lesion length on the leaves of the mutant lines was 0.5 ± 0.27 cm and was shorter than that on the leaves of segregate wild type (*Se-WT_dj_*) (9.6 ± 1.04 cm) (**Figures 1C,D**). The population of bacteria extracted from the inoculated leaves was significantly reduced in the mutant rice leaves compared to that extracted from the inoculated *Se-WT_dj_* leaves (**Figure 1E**). These results indicate that rice lacking the *Os01g02290* gene shows strong resistance against PXO99. The enhanced resistance against PXO99 was confirmed in the other mutant line, 3B-00367 (named Δ*rrsRLK*_hy_ in this study). Expression of the gene was inhibited by T-DNA insertion at the first exon (nt 581) of *Os01g02290* in Hwayoung rice (Supplementary Figures S1A,B). From the inoculation test with HB01009 (*Xoo* Korean strain 3a, compatible to Hwayoung), lesion lengths on leaves of the Δ*rrsRLK*_hy_ rice were reduced about 71.8% (3.13 ± 1.37 cm) compared to those on the leaves of segregate wild type (*Se-WT_hy_*) (11.11 ± 1.04 cm) (Supplementary Figures S1C,D). We also found gene activation lines (4A-01523 and 2A-50012) in which expression of *Os01g02290* was enhanced by T-DNA insertion at nt 6877 (named *rrsRLK_act1_*) and nt 4068 (named *rrsRLK_act2_*), respectively (Supplementary Figures S1E,F). However, lesion lengths on the leaves of the activation lines were not significantly different from those of *Se-WT_dj_* (Supplementary Figures S1G,H).

### Δ*rrsRLK*_dj_ Has a Broad Range of Resistance Against *Xoo* Strains, but Not to the Fungal Pathogen *M. grisea*

To test if the resistance of the mutant is specific to the PXO99 strain, another Philippine strain, PXO86, and Korean strains (HB01009, HB01013, HB01014, HB01015) were inoculated on leaves of the Δ*rrsRLK*_dj_ and *Se-WT_dj_* lines. All five strains are known to be compatible with Dongjin. Inoculation of each strain caused long lesion development on the leaves of *Se-WT_dj_* rice, while lesion lengths on the leaves of Δ*rrsRLK*_dj_ were dramatically reduced (Supplementary Figure S2A). The results suggest that absence of the *Os01g02290* gene in rice induces a broad range of resistance to *Xoo* strains. To study the possible resistance of Δ*rrsRLK*_dj_ against fungal pathogens, an inoculation test with *M. grisea* (compatible with Dongjin) was carried out. In the test, leaves of the Δ*rrsRLK*_dj_ and *Se-WT_dj_* rice showed similar lesion lengths (Supplementary Figure S2B), indicating that knock-out of the *Os01g02290* gene does not induce resistance against the fungal pathogen *M. grisea*.

### Suppression of *Os01g02290* Gene Expression Induces Semi-Dwarfism and Lesion Mimic Phenotypes

**FIGURE 2:**
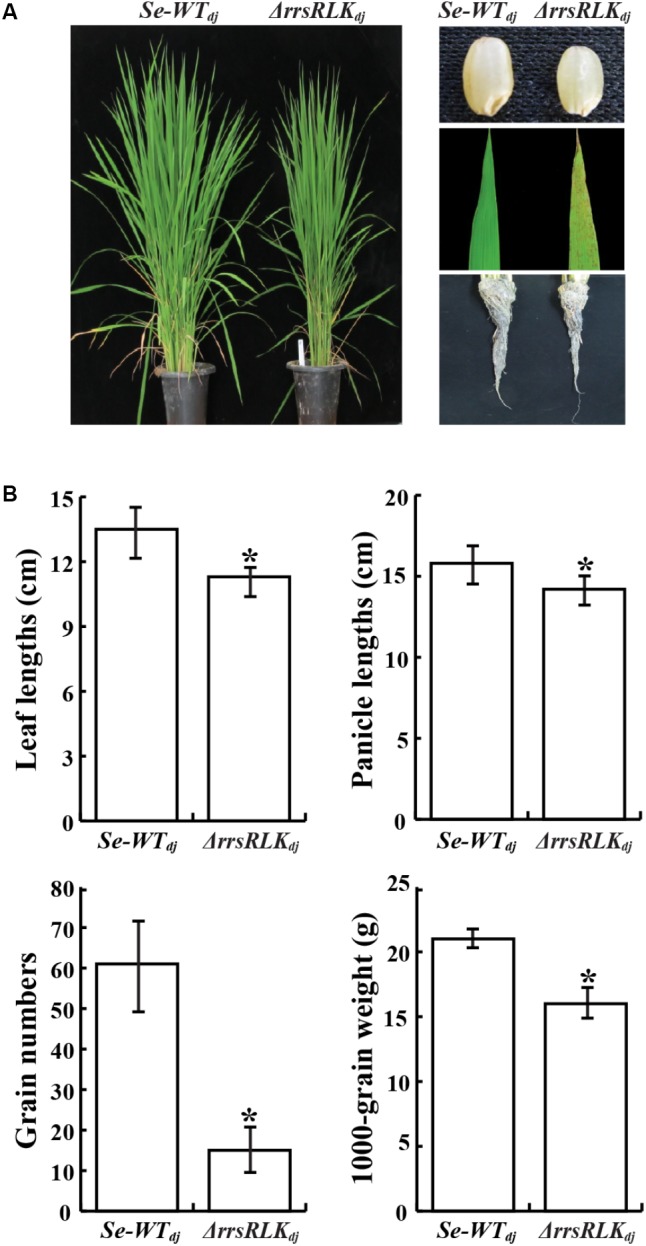
**(A)** Comparison of morphology between *SE-WT_dj_* and homozygous lines of Δ*rrsRLK*_dj_. Leaf lesions mimic symptoms and grain phenotypes of *SE-WT_dj_* and Δ*rrsRLK*_dj_ (Right). **(B)** Phenotypic data compared between *SE-WT_dj_* and Δ*rrsRLK*_dj_ (leaf and panicle lengths, grain numbers and weight) from the T3 generation grown in a paddy field were collected in 2015. Asterisks indicate *P* < 0.05 (Student’s *t*-test).

Morphological changes of Δ*rrsRLK*_dj_ were monitored through the sixth generation. We found that Δ*rrsRLK*_dj_ displayed semi-dwarfism. At the 8-leaf stage, the Δ*rrsRLK*_dj_ showed a 10% reduction in rice height compared to *Se-WT_dj_* in paddy fields (**Figures 2A,B**). The seeds of Δ*rrsRLK*_dj_ were smaller in panicle length, weight, and height to those of *Se-WT_dj_* (**Figures 2A,B**). The semi-dwarfism was also shown in roots (**Figure 2A**). In particular, Δ*rrsRLK*_dj_ had about a 75% decrease in grain numbers compared to *Se-WT_dj_* (**Figure 2B**). Additional changes include reddish brown spot lesions scattered across the entire surface of the leaves, with severity increasing at the tips (**Figure 2A**). However, the lesion-mimic phenotype on the mutant leaves appeared conditionally; it only appeared on rice grown in the paddy field during the summer season. All of the changes in rice height and seed size that were observed in Δ*rrsRLK*_dj_ were similarly observed with Δ*rrsRLK*_hy_, including the lesion-mimic. These results suggest that the rrsRLK protein from the *Os01g02290* gene contributes to both vegetative and reproductive stages in rice. In addition, the lesion-mimic on the mutant leaves, which only appeared during the summer, leads us to suspect that the protein may have a function associated with tolerance under the conditions.

### *Os01g02290.2* Encoding a Tyr Receptor-Like Kinase Is Only Expressed in Rice

According to the rice genome database^2^, two genetic forms of *Os01g02290* can be expressed by alternative splicing. Per annotation of the database, *Os01g02290.1* encodes a Ser/Thr receptor-like kinase, and the alternative form *Os01g02290.2* encodes a Tyr receptor-like kinase. To determine the expression of both forms, RT-PCR was carried out with specific primers (Supplementary Table S4) and RNAs extracted from each leaf stage of Dongjin and Hwayoung. As shown in Supplementary Figure S3A, the alternative splicing form 1 (*Os01g02290.1*) did not amplify at any stage, while *Os01g02290.2* was expressed at all stages in both rice lines, though it was slightly decreased after the six-leaf stage in both lines. From additional RT-PCR to test tissue specificity of the gene expression, *Os01g02290.1* did not appear in any tissue, while *Os01g02290.2* was expressed in all tissues. The highest expression was seen in the root of both wild types (Supplementary Figure S3B), while the seed showed very slight expression. These results suggest that only *Os01g02290.2* annotated to encode a Tyr receptor-like kinase is functional in rice, and the protein dominantly contributes at the vegetative phase and in the root.

*Os01g02290.2* consists of four exons split by three introns. The domain structure of the protein deduced from the DNA sequence of *Os01g02290.2* is predicted to have a signal peptide (aa 1–21), a transmembrane (aa 263–285), and a tyrosine kinase (aa 325–393) (Sakai et al., 2013^3^). To determine if the kinase domain phosphorylates tyrosine residue in a substrate protein, a recombinant protein with the kinase domain tagged with 6x Histidine at the N-terminus was generated (Supplementary Figure S3C) and used for the tyrosine kinase activity assay. As shown in Supplementary Figure S3D, the recombinant protein showed significant tyrosine kinase activity, as seen in the control, c-Src (Proto-oncogene tyrosine-protein kinase), while the protein extract from *Escherichia coli* carrying only the pET28-a vector did not. This result reveals that the domain of the protein encoded by the *Os01g02290.2* gene has kinase activity that phosphorylates a tyrosine(s) of a target protein.

### Lack of *rrsRLK* Induces Expressional Changes of *PR* and Phytohormone-Related Genes

**FIGURE 3:**
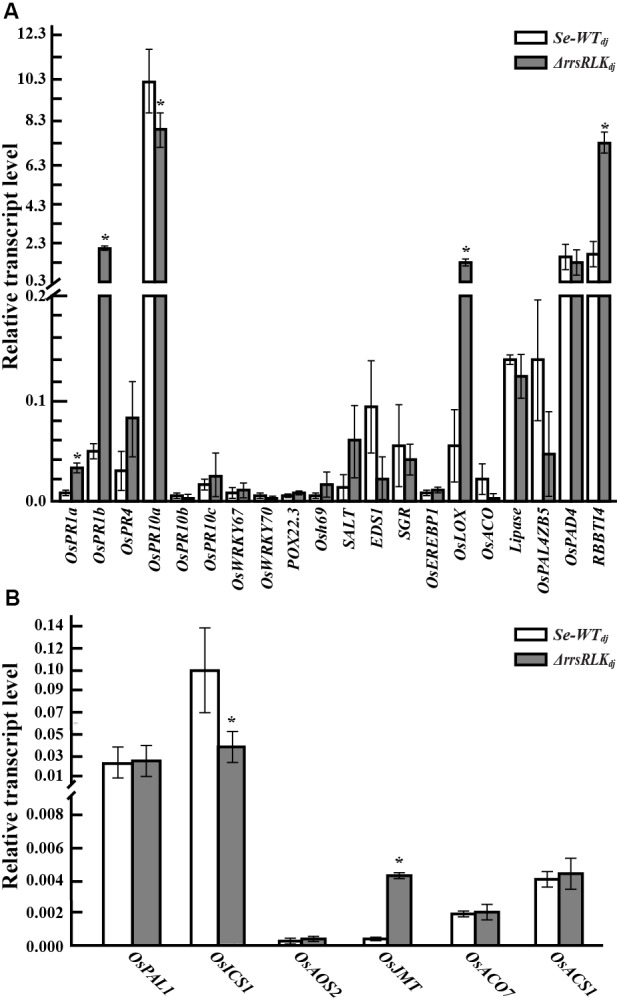
The expression analysis of pathogen-related **(A)** and hormone-related genes **(B)** in rice plants grown for 6 weeks. RNA for qRT-PCR was extracted from leaves of 6-week-old *SE-WT_dj_* and Δ*rrsRLK*_dj_ mutant lines. Data represent mean ± SD of three independent measurements. Asterisks indicate statistical significance according to Student’s test; ^∗^*P* < 0.05.

Many pathogenesis-related (*PR*) and phytohormone-related genes involved in plant defense have been reported (Agrawal et al., 2000; Bari and Jones, 2009). To test if absence of the *rrsRLK* gene has an effect on expression of *PR* and hormone-related genes, qRT-PCR analysis was carried out for 20 *PR* and six hormone-synthesis genes (listed in Supplementary Table S3) in *Se-WT_dj_* and Δ*rrsRLK*_dj_. From the analyses, we found that expression of *OsPR1a*, *OsPR1b*, *OsLOX*, and *RBBTI4* was significantly increased in Δ*rrsRLK*_dj_, and *OsPR10a* was decreased in Δ*rrsRLK*_dj_ (**Figure 3A**). The *RBBTI4* gene encodes a trypsin inhibitor and is associated with immunity in animals, plants, and microbes (Farmer and Ryan, 1990; Song et al., 1999; Meyskens et al., 2001). Although it was not tested using purified RBBTI4 protein, inhibitory activity against trypsin in total protein extract from Δ*rrsRLK*_dj_ was significantly increased compared to that from *Se-WT_dj_* (Supplementary Figure S4).

In the test with phytohormone-related genes involved in salicylic acid (SA), jasmonic acid or methyl-jasmonic acid (JA or MeJA), and ethylene (ET) synthesis, expression of the *OsICS1* gene in Δ*rrsRLK*_dj_ was significantly decreased, and expression of the methyl-jasmonate biosynthesis (*OsJMT*) gene in Δ*rrsRLK*_dj_ was significantly increased (**Figure 3B**). In ET-related genes, we did not observe any change in expression. These results reveal that deficiency of *rrsRLK* induces *PR* gene expression including *OsPR1a*, *OsPR1b*, *OsLOX*, and *RBBTI4*, as well as JA/MeJA synthesis.

### The rrsRLK Protein Localizes in Cytoplasm, Not in the Plasma Membrane or Nuclei

**FIGURE 4:**
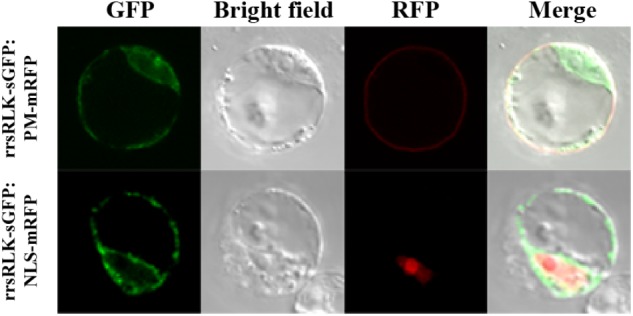
Sub-cellular localization of the rrsRLK protein in rice cells. The rrsRLK-GFP fusion protein was transiently expressed in OC cells and monitored with a confocal microscope equipped with filter sets for GFP (excitation wavelength/dichroic transition: 488/543 nm) and RFP (excitation wavelength/dichroic transition: 561/575 nm). GFP, OC cells showing rrsRLK-GFP green fluorescence in cytosol; RFP, the same cell showing plasma membrane and nuclear localization using PM-mRFP and NLS-mRFP markers (Kim et al., 2009); Bright field, a differential interference contrast image; Merged, the merged image of GFP and RFP.

The domain structure of rrsRLK protein suggests that it might be localized in the plasma membrane. To determine the subcellular localization of the protein, a construct to generate rrsRLK protein fused by GFP at the C-terminus was introduced in OC cells, and the GFP signal was monitored (**Figure 4**). In this transient expression assay, the rrsRLK-GFP signal was only detected in cytoplasm and was not merged with any signal from the nuclear marker NLS-mRFP or the plasma membrane marker PM-mRFP (**Figure 4**). This result indicates that rrsRLK is a cytoplasmic protein, not localized in the plasma membrane or nuclei.

### The rrsRLK Protein Interacts With OsPEX11 and OsVOZ1

**FIGURE 5:**
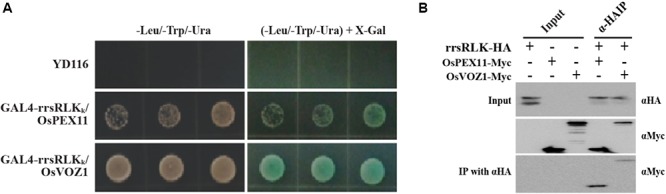
Interaction between rrsRLK, OsPEX11, and OsVOZ1. **(A)** Yeast two-hybrid assay carried out with rrsRLK_k_ as a bait protein. The plasmids (pD32rrsRLK_k_ and pD22Lib) were co-transformed into the yeast strain YD116. The transformants were grown on selective minimal medium without Leu, Trp, and Ura. The interaction was confirmed by measuring the activity of β-galactosidase in yeast. **(B)** Co-immunoprecipitation assay of rrsRLK, OsVOZ1, and OsPEX11. A combination of the constructs was transformed into rice protoplasts for transient expression and immunoprecipitated with anti-HA (αHA) or anti-Myc (αMyc) antibodies. The immunoprecipitates were analyzed with a Western blot with anti-HA or anti-Myc antibodies. 10% of the pre-cleared extracts were used as input controls.

To identify interacting protein(s) downstream of rrsRLK-mediated signaling, we carried out a yeast two-hybrid assay using GAL4-rrsRLK_k_ (rrsRLK kinase domain, aa 325–393) as a bait protein and a rice cDNA library. We found eight interacting candidates: *O. sativa* vascular plant one zinc finger protein 1 (OsVOZ1: Os01g0753000), *O. sativa* peroxisomal biogenesis factor 11 (OsPEX11: Os03g0117100), UMP synthase (Uridine 5′-monophosphate synthase), Chlorophyll A-B binding protein, Dehydrogenase, and three drought-induced proteins. In order to confirm the binding abilities of these candidate proteins, X-gal blue/white selection was carried out (**Figure 5A**). Only two of the proteins, OsVOZ1 and OsPEX11, showed positive signals. The interactions between full-length rrsRLK and the two proteins were confirmed by a coimmunoprecipitation assay in which the former was tagged with HA (rrsRLK-HA) and the latter with Myc (OsVOZ1-Myc and OsPEX11-Myc) (**Figure 5B**). The tagged proteins were co-expressed in rice mesophyll protoplasts using the maize Ubiquitin1 promoter. Analysis with anti-HA and anti-Myc antibodies showed that rrsRLK truly interacts with OsVOZ1 and OsPEX11, respectively.

### Lack of OsVOZ1 and OsPEX11 Induced Resistance Against *Xoo* in Rice

**FIGURE 6:**
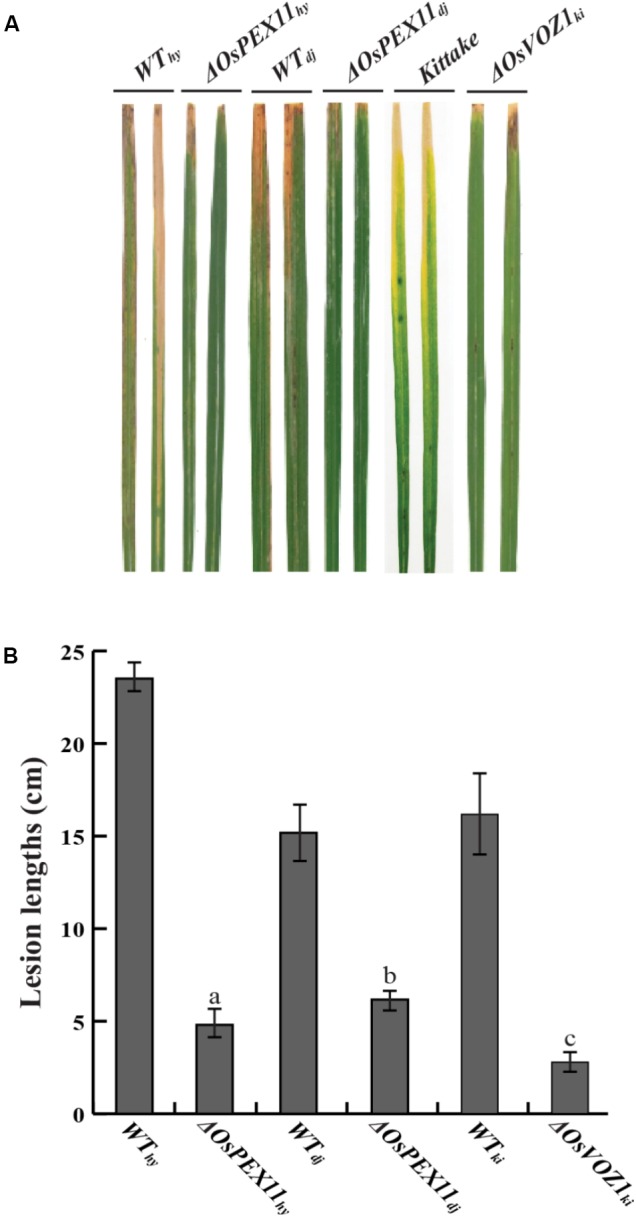
Inoculation assay with *OsPEX11* (Δ*OsPEX11*_hy_ and Δ*OsPEX11*_dj_) and *OsVOZ1* (Δ*OsVOZ1*_ki_) gene knock-out mutant lines. **(A)** *Xoo*-inoculated leaves of wild type rice (Hwayoung, Dongjin, and Kittake) and homozygous line of OsPEX11 and OsVOZ1. Each line was inoculated with *Xoo* strains (PXO99 for Dongjin and Kittake and HB01009 for Hwayoung) using a clipping method, and the lesion development on the leaves was monitored for 14 DAI. **(B)** Lesion lengths of the wild type and mutant rice leaves. The lesion lengths were scored at 14 DAI. The averages and error ranges of each data set were calculated from 40 leaves. The experiment was repeated three times with consistent results. Different letters above bars indicate statistically significant differences as determined by one-way analysis of variance (ANOVA: a, b, c), *P* < 0.05.

Identification of the two interacting proteins led us to hypothesize that OsVOZ1 and/or OsPEX11 might be signaling partners, and that signaling is negative for *Xoo*-resistance. To test this hypothesis, we selected T-DNA insertion mutants for *OsPEX11* (1B-07040 and 1B-03689) and *OsVOZ1* (K-05631) from the rice T-DNA mutant population. 1B-07040 has a T-DNA insertion at the first exon of *Os03g0117100* encoding OsPEX11 in Dongjin (Δ*OsPEX11*_dj_), and the 1B-03689 line has a T-DNA insertion 100 bp upstream from the end of the sixth exon of the gene in Hwayoung (Δ*OsPEX11*_hy_) (Supplementary Figure S5A). In K-05631, T-DNA is inserted 259 bp upstream from the start codon of Os01g0753000 encoding OsVOZ1 in Kittake (Δ*OsVOZ1*_ki_) (Supplementary Figure S5B). Expression of the *OsPEX11* in 1B-07040 and 1B-03689 and *OsVOZ1* in K-05631 was tested with RT-PCR (Supplementary Figures S5C–E) and T2 generation of the mutant rice plants inoculated by *Xoo* strains [PXO99 for Δ*OsPEX11*_dj_ and Δ*OsVOZ1*_ki_, and HB01009 for Δ*OsPEX11*_hy_]. All lesion lengths scored at 14 days after inoculation were dramatically shortened compared to each wild type (**Figures 6A,B**). Lesion lengths on leaves of the Δ*OsPEX11*_hy_ (4.8 ± 1.50 cm) and Δ*OsPEX11*_dj_ (4.6 ± 1.59 cm) strains were reduced by 64.7 and 79.58% compared to the ones of Hwayoung (23.5 ± 1.96 cm) and Dongjin (14.8 ± 2.78 cm), respectively. Lesions on the leaves of the Δ*OsVOZ1*_ki_ (3.9 ± 1.25 cm) line were reduced by about 65% compared to the ones of Kittake (15.85 ± 2.19cm). These shorter lesion lengths reveal that signals caused by interactions between rrsRLK and the two proteins have negative effects on resistance to *Xoo* in rice.

### H_2_O_2_ Is Accumulated in Δ*rrsRLK* Rice Due to a Decrease in Activities of ROS-Scavenging Enzymes

**FIGURE 7:**
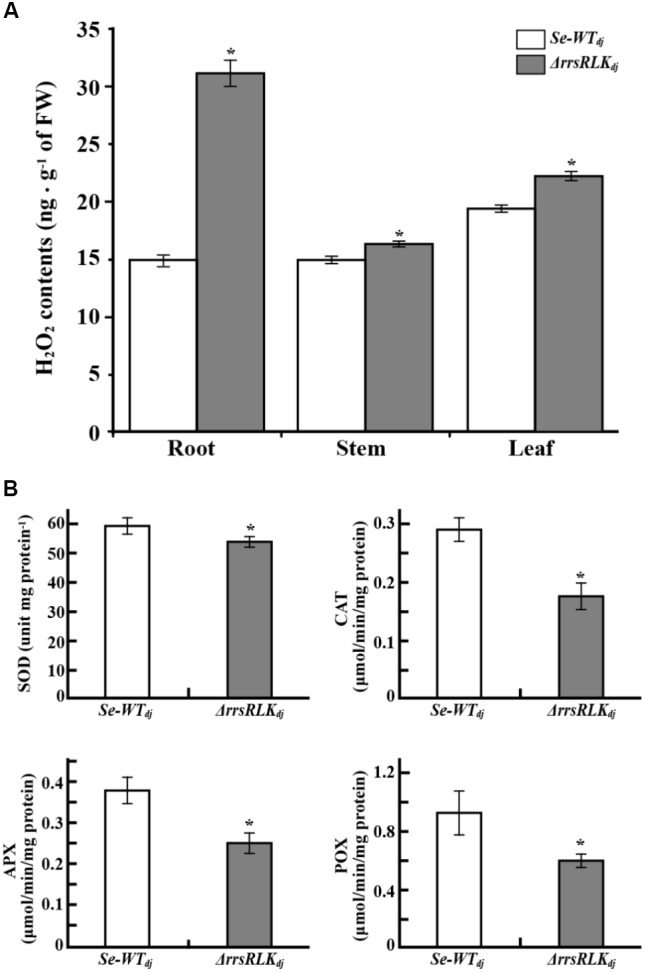
**(A)** H_2_O_2_ content in tissues of *SE-WT_dj_* and Δ*rrsRLK*_dj_. Content of H_2_O_2_ in 1 g of tissue (root, stem, and leaf) of *SE-WT_dj_* and Δ*rrsRLK*_dj_ was spectrophotometrically determined at 436 nm by the modified method of Bernt and Bergmeyer (1974) using peroxidase enzyme. **(B)** Activity assay of superoxide dismutase (SOD), catalase (CAT), ascorbate peroxidase (APX), and peroxidase (POX) in leaves of *SE-WT_dj_* and Δ*rrsRLK*_dj_. Total protein was extracted from 1 g of leaves from *SE-WT_dj_* and Δ*rrsRLK*_dj_ rice, and the activity of ROS-scavenging enzymes was spectrophotometrically monitored at A_560nm_ (SOD), A_240nm_ (CAT), A_290nm_ (APX), and A_436nm_ (POX) using a modified method of Beyer and Fridovich (Beyer and Fridovich, 1987). All assays were repeated more than three times, and the error bars of each sample denote the mean ± SD (*n* = 9). The asterisks indicate that a significant difference (*P* < 0.05) in the H_2_O_2_ was detected between transgenic plants and wild type.

PEX11 is an essential protein for peroxisome multiplication (Erdmann and Blobel, 1995; Marshall et al., 1995), and peroxisomes are responsible for detoxification of ROS. The results described above suggest that Δ*rrsRLK* might have abnormal ROS contents. We investigated H_2_O_2_ contents in the roots, stems, and leaves of Δ*rrsRLK*_dj_ and compared to those of wild type. H_2_O_2_ contents were increased in all of the tissues of Δ*rrsRLK*, particularly in the roots (**Figure 7A**). Accumulation of H_2_O_2_ in Δ*rrsRLK*_dj_ was also investigated in an experiment using DAB staining. As shown in Supplementary Figure S6A, accumulation of H_2_O_2_ in the leaf, stem, and root of the Δ*rrsRLK*_dj_ was clearly detected. Using a higher magnification than in Supplementary Figure S6A, we noted that the accumulation of H_2_O_2_ was higher in cells of Δ*rrsRLK*_dj_ leaf. In addition, Δ*rrsRLK*_dj_ cells had giant spots that seem to be peroxisomes, while the wild type has scattered small spots (Supplementary Figure S6B). These results suggest that rrsRLK contributes to regulation of the amount of H_2_O_2_ in rice. Furthermore, we tested gene expression of burst oxidase homologs (*RBOHs*), because additional ROS production in apoplast occurs through the protein (Kadota et al., 2014). However, rice *RBOH* genes showed no changes in expression (Supplementary Figure S7). Based on the results, we finally carried out experiments to test gene expression against most of available genes (around 80 genes) encoding ROS-scavenging enzymes and to measure the activity of ROS-scavenging enzymes, SOD, CAT, APX, and POX. Among the genes that we tested, *Os03g11960* encoding Cu-Zn SOD, *Os02g02400* encoding Cat, *Os06g37150* encoding L-APX, and *Os07g48020* encoding POX were representatively reduced in expression as shown in Supplementary Figure S8. The activity of SOD, CAT, POX, and APX in protein extract from the mutant rice leaves were significantly decreased in Δ*rrsRLK*_dj_ compared to Dongjin (**Figure 7B**).

## Discussion

In higher plants as well as in animals, ROS (e.g., O_2_^⋅-^, H_2_O_2_, OH^⋅^, ^1^O_2_) have highly versatile properties. In early studies, ROS were regarded as toxic byproducts in plants, continuously produced through aerobic metabolism such as respiration and photosynthesis. It is now understood that ROS play beneficial roles in most cellular processes. The differentiation of plant tissues is characterized by ROS homeostasis, especially in roots. UPB1, a member of the basic/helix loop-helix (bHLH) TF family, is a well-known factor of root differentiation acting by controlling peroxidases expression (Tsukagoshi et al., 2010). ROS have been also identified as essential partners of auxin-signaling for gravitropism of maize root (Joo et al., 2001) and participate in rice seed germination associated with ABA (Ye et al., 2011). In addition to the evidence that ROS contribute to plant development, it has been suggested that they also play a role as rapidly generated signal molecules in response to stresses (Stael et al., 2015). Perception of PAMPs by RLKs immediately induces additional ROS production (Husi et al., 2000; Mersmann et al., 2010; Petutschnig et al., 2010) in apoplasts by nicotinamide adenine dinucleotide phosphate (NADPH) oxidases (often called respiratory burst oxidase homologs, RBOHs) as a part of the PRR complex (Kadota et al., 2014). Both expression of *Rboh* genes and activation of the proteins are triggered by PAMP recognition (Ogasawara et al., 2008; Boudsocq et al., 2010; Morales et al., 2016). The additional ROS in apoplasts enter the cell via aquaporin to alter cellular processes such as activation of defense responses, regulation of photosynthesis, modulation of hormonal responses, and inhibition of growth and development (Mittler and Blumwald, 2015). These alterations are eventually terminated by reduction of ROS to appropriate levels by scavenging enzymes. APX, SOD, POX, and CAT are the enzymes responsible for ROS homeostasis (Mittler et al., 2004). Our results showed that H_2_O_2_ content is significantly increased in Δ*rrsRLK* regardless of pathogen infection (**Figure 7A** and Supplementary Figure S6). H_2_O_2_ accumulation in apoplasts of the Δ*rrsRLK*_dj_ leaves was also observed (Supplementary Figure S6B). However, H_2_O_2_ accumulation in the tissues and apoplasts of Δ*rrsRLK* is not related to additional production by RBOHs because no change of *Rboh (A, B, C, D, E, F, G, I)* gene expression was observed in the mutant (Supplementary Figure S7). Although we cannot explain why more H_2_O_2_ was observed in the apoplasts of Δ*rrsRLK*, it is clear that abnormal ROS levels conferring broad spectrum resistance to *Xoo* are due to irregularity of the scavenging system caused by a reduction of scavenging-enzyme activity in the Δ*rrsRLK* lines (**Figure 7B**). However, we suspect that H_2_O_2_ level in Δ*rrsRLK* rice is not sufficient to enhance resistance to *M. grisea*, although it is resistant to *Xoo*. Around 10 times higher ROS amount by inhibition of H_2_O_2_ degradation in *Bsr-d1* knock-out rice than wild type confers blast resistance (Li et al., 2017). Compared to the mutant, Δ*rrsRLK* rice possesses maximal two times higher H_2_O_2_ in root, and the increases in stem and leaf were smaller than that in root. Under consideration of H_2_O_2_-sensitivity of bacteria and fungi (Hong et al., 2013), this possibly explains why Δ*rrsRLK* rice has resistance to only the bacterial pathogen.

In our experiments, yeast two-hybrid assays and coimmunoprecipitation tests resulted in identification of two interacting proteins (**Figure 5**). The knock-out mutants of genes encoding the interacting proteins showed strong resistance to *Xoo* (**Figure 6**). One of the two proteins, OsPEX11, is essential in plants for peroxisome multiplication. Peroxisomes are derived from the endoplasmic reticulum and are created by division of pre-existing peroxisomes (Tabak et al., 2006). One of the important roles of plant peroxisomes is detoxification of cells by decreasing H_2_O_2_ levels produced by chloroplasts and mitochondria in photorespiration. Since the first peroxisome division protein, Sc-PEX11, was identified in yeast (Erdmann and Blobel, 1995; Marshall et al., 1995), homologs of Sc-PEX11 as well as many proteins involved in peroxisome biogenesis have been reported in *Arabidopsis* (McCartney et al., 2005). Ectopic expression of *Sc-PEX11* caused organelle elongation/tubulation for multiplication, and the null mutant of the gene showed one or two giant peroxisomes in cells (Erdmann and Blobel, 1995; Marshall et al., 1995), as shown in our results (Supplementary Figure S6B). Lesion mimic under summer conditions (**Figure 2**) can be explained by this. Vigorous H_2_O_2_ production under the summer conditions is beyond the detoxifying ability in Δ*rrsRLK* mutant rice. These changes in Δ*rrsRLK* clearly illustrate the rrsRLK function in rice biology as a regulator for peroxisome multiplication and ROS homeostasis.

The VOZ protein was reported as a novel transcription factor in *A. thaliana* (Mitsuda et al., 2004). AtVOZ interacts with phytochrome B and accelerates flowering time in *A. thaliana* (Yasui et al., 2012). AtVOZ2 is controlled by light quality in a phytochrome-dependent manner (Yasui et al., 2012). Loss of function in *voz* genes enhanced resistance to freezing, cold, and drought, suggesting that it acts as a negative regulator for resistance against abiotic stresses. VOZs also act as a positive regulator for resistance to the fungal pathogen because the *voz1voz2* mutant showed increased susceptibility for *Colletotrichum higginsianum* (Nakai et al., 2013). Rice cultivar Dongjin has two orthologs of AtVOZ2, OsVOZ1, and OsVOZ2, with 60.4% identity in amino acid sequences. Interestingly, *OsVOZ2* knock-out mutant rice showed enhanced resistance (Cheong et al., 2013). In the report, OsVOZ2 is suggested as a target protein of the *Xoo* type-three effector XopN_KX085_, and the interaction between OsVOZ2 and XopN_KX085_ is important for *Xoo* virulence. This means that OsVOZ2 is a negative regulator for gene expression responsible for defense against *Xoo*. Although the report concluded that OsVOZ1 is not a target of XopN_KX085_, our results demonstrate that OsVOZ1 negatively functions in the expression of defense-related genes in rice (**Figure 6**) and is regulated by interaction with rrsRLK.

Over the past several decades, PR proteins have been reported in a wide variety of plant species (Linthorst and Van Loon, 1991). In our experiments, Δ*rrsRLK*_dj_ mutant lines showed expression changes of various *PR* genes. Expression of *OsPR1a*, *OsPR1b*, *OsLOX*, and *RBBTI4* was increased in Δ*rrsRLK*_dj_, while that of *OsPR10a* was decreased (**Figure 3A**). *PR1* genes are often used as a marker for studying resistance in plants. In rice, 32 *PR1* genes are predicted in the rice genome (van Loon et al., 2006). Among them, *OsPR1a* and *OsPR1b* are relatively well studied. These two *PR1* genes are known to respond to fungal infection (Agrawal et al., 2001), environmental stresses (Agrawal et al., 2000, 2001), and chemical treatment (Malamy et al., 1990). The expression of *OsPR1* genes is generally implicated in the antagonistic relationship between SA and JA (Mitsuhara et al., 2008), and SA-JA antagonism is likely conserved in rice (Yuan et al., 2007). However, the signaling pathways of SA and JA are often documented as synergistic (Xie et al., 2011). *OsLOX* is a JA biosynthesis gene in rice (Liu et al., 2012). JA biosynthesis requires three rounds of peroxisomal β-oxidation for the conversion of OPDA (12-Oxo-Phytodienoic Acid) to JA (Vick and Zimmerman, 1984; Dave and Graham, 2012). In our experiments, deficiency of *rrsRLK* induced gene expression of *OsLOX* and *OsJMT* (**Figure 3**). This suggests that rrsRLK or a signal mediated by rrsRLK has a negative effect on JA or MeJA synthesis. Expression of the *OsPR10a* gene is induced by pathogens including *Xoo* (Ryu et al., 2006) and diverse stimuli such as SA, JA, ethephone, NaCl, and ABA (Hwang et al., 2008). Although our test results demonstrated that SA and JA have an antagonistic relationship in Δ*rrsRLK*_dj_ (**Figure 3B**), it is possible that *OsPR10a* expression is dominantly affected by the SA signaling in rice. *RBBTI* genes encode a cysteine-rich serine protease inhibitor in rice (Pang et al., 2013). There is a lot of evidence that protease inhibitors are inducible in pathogen attack (Roby et al., 1987; Rickauer et al., 1989; Cordero et al., 1994; McGurl et al., 1994; Kim and Choi, 2000; Pernas et al., 2000) and contribute to resistance against pathogenic proteases by inhibition (Peng and Black, 1976; Yamaleev et al., 1980; Brown and Adikaram, 1983). Bacterial proteases are one of the (a)virulence factors targeting host proteins. For instances, YopJ of *Yersinia pestis* and XopD of *Xanthomonas campestris* pv. *vesicatoria* are cysteine proteases that alter the sumoylation process by targeting SUMO (small ubiquitin-like modifier) proteins to inhibit multiple signaling (Orth et al., 2000; Hotson et al., 2003). On the other hand, host plants have developed appropriate defense measures, such as BBTI, to suppress the activity of proteases from pathogens. Since the first identification of soybean BBTI proteins (Bowman, 1946; Birk, 1961; Birk et al., 1963), homologs have been identified in many plants and their functions in the biology of plants and their interactions with microbial invaders studied (Haq et al., 2004; Yan et al., 2009). In rice, overexpression of RBBI2-3 and BBTI4 confers resistance to rice blast and bacterial leaf blight (Pang et al., 2013), respectively. Our results agree with the previous report: induced RBBTI4 expression in Δ*rrsRLK* mutant lines (**Figure 3**) showed resistance against *Xoo* strains. Additionally, our results confirm that expression of the *RBBTI4* gene is also related to hormone signaling (Rakwal and Komatsu, 2000). Exogenous JA treatment results in reduction of ribulose-1, 5-bisphosphate carboxylase/oxygenase subunits and induction of novel proteins, including RBBTI4. This suggests that induction of RBBTI4 expression is positively regulated by JA signaling. Together, these findings suggest that the expressional change of *PR* genes in Δ*rrsRLK* is due to change of phytohormones synthesis, particularly of JA induced by H_2_O_2_ accumulation in Δ*rrsRLK* rice. ROS signals interact with several other signaling pathways such as nitric oxide and the stress hormones salicylic acid, jasmonic acid, and ethylene (Gechev et al., 2006). Their inter-relationships are very complex, and many questions about these relationships have yet to be answered. Hu et al. (2003) reported that H_2_O_2_ stimulates JA accumulation, but JA has no effect on H_2_O_2_ generation in suspension-cultured cells of Panax ginseng. Zhao and Sakai (2003) observed that H_2_O_2_ treatment stimulates activity of LOX, important for the biosynthesis of jasmonate in *Cupressus Lusitanica* cell cultures. Furthermore, a conditional oxidative stress-signaling mutant of *Arabidopsis*, which shows H_2_O_2_-activated oxidation and glutathione accumulation, accumulates transcripts of the four genes *LOX3*, *OPR3*, *JAZ10*, and *VSP2* involved in JA synthesis and signaling, suggesting that intracellular oxidative stress activates JA signaling (Han et al., 2013).

Based on our data, deficiency of *rrsRLK* in rice causes H_2_O_2_ accumulation by abnormal peroxisome multiplication and reduction of activity of ROS-scavenging enzymes. This leads to an increase in a phytohormone such as JA (MeJA) and an induction of *PR* gene expression. Although questions remain to be answered, if rrsRLK interacts with an effector from *Xoo* and/or recognizes an internal molecule as a signal to modulate ROS level in rice, we conclude that rrsRLK is one of key regulators for ROS homeostasis and plays a negative role in rice defense against the bacterial pathogen *Xoo*.

## Author Contributions

S-WL conceived and designed the experiments. YY, J-CP, JY, C-YK, M-HC, K-HJ, J-SJ, and GA performed the experiments and conducted bioinformatics analyses. YY, J-CP, and S-WL analyzed the data and wrote the manuscript.

## Conflict of Interest Statement

The authors declare that the research was conducted in the absence of any commercial or financial relationships that could be construed as a potential conflict of interest.

## Footnotes

1 http://www.dna.affrc.go.jp/

2 http://rice.plantbiology.msu.edu

3 http://smart.embl-heidelberg.de/
