# Supplementary material for: Lack of a Cytoplasmic RLK, Required for ROS Homeostasis, Induces Strong Resistance to Bacterial Leaf Blight in Rice
Source: Front Plant Sci. 2018 May 18;9:577. doi: 10.3389/fpls.2018.00577 (PMC5968223; doi:10.3389/fpls.2018.00577)
Supplement: Supplementary file 1 [file Table_1.docx]

Table S1. Plants, bacterial and fungal strains used in this study

| **Plant** | **Background** | | **Selectable marker** | **Reference** |
| --- | --- | --- | --- | --- |
| 3A-10392 | Variety: Dongjin, Insertion position: 719607bp | Hygromycin | | Jeon *et al*., 2000 |
| 4A-01523  2A-50012  3B-00367  K-05631 | Vector: pGA2715  Variety: Dongjin, Insertion position: 713208bp  Vector: pGA2715  Variety: Dongjin, Insertion position: 716017bp  Vector: pGA2715  Variety: Hwayoung, Insertion position:720302bp  Vector: pGA2707  Variety: Kittake, Insertion position: 3157326bp  Vector: pGA2715 | Hygromycin  Hygromycin  Hygromycin  Hygromycin | | Jeon *et al*., 2000  Jeon *et al*., 2000  Jeon *et al*., 2000  Jeon *et al*., 2000 |
| 1B-07040  1B-03689 | Variety: Dongjin, Insertion position: 938255bp  Vector: pGA2715  Variety: Hwayoung, Insertion position:939676bp  Vector: pGA2715 | Hygromycin  Hygromycin | | Jeon *et al*., 2000  Jeon *et al*., 2000 |

| **Strain** | **Characteristics** | **Reference** |
| --- | --- | --- |
| HB01013  (*Xoo* Korea race 1) | wild-type, Korean race 1 strain that is virulent to rice carrying the *Xa10*, *Xa21* resistance gene, Ceph^R^ | Lee *et al*., 2005 |
| HB01014  (*Xoo* Korea race 2) | wild-type, Korean race 1 strain that is virulent to rice carrying the *Xa1, Xa10, Xa11, Xa13* resistance gene, Ceph^R^ | Kim *et al*., 2009 |
|  |  |  |
| HB01015, HB01009  (*Xoo* Korea race 3, 3a) | wild-type, Korean race 1 strain that is virulent to rice carrying the *Xa1, Xa2, Xa8, Xa10, Xa11, Xa13* resistance gene, Ceph^R^ | Kim *et al*., 2009 |
| PXO99az | Wild-type, Philippine race 6, NC_010717 | Bogdanove *et al*., 2011 |
| DH5α | F^-^ Φ80d*lacZ*Δ*M15* Δ(*lacZYA-argF* )*U169 endA1 recA1 hsdR17* (r_K_^-^m_K_^+^) *deoR thi-1 supE44 λ-gyrA96 relA1* | Gibco BRL |
| YD116 | MATa ura3-52 his3-200 leu2- trp1-901 canR gal4Δ512 gal80 Δ 338 lys2- 801::UASG–HIS3-lacZ ade2-101::GAL1-URA3 | Invitrogen |
| PO6-6 | Philippine race | Lee *et al*., 2005 |
| (Magnaporthe grisea) |  |  |

Cm^R^, chloramphenicol resistance; Sp^R^, spectinomycin resistance; Tet^R^, tetracycline resistance; Amp^R^, ampicillin resistance; Nm, neomycin
