# Supplementary material for: Lack of a Cytoplasmic RLK, Required for ROS Homeostasis, Induces Strong Resistance to Bacterial Leaf Blight in Rice
Source: Front Plant Sci. 2018 May 18;9:577. doi: 10.3389/fpls.2018.00577 (PMC5968223; doi:10.3389/fpls.2018.00577)
Supplement: Supplementary file 2 [file Table_2.DOCX]

Table S2. Primers used for determination of the genotype of each rice line

| **Gene** | **Primer sequence** |
| --- | --- |
| *ΔrrsRLK_dj_* -F  *ΔrrsRLK_dj_* -R | 5’-TGGAAGTAAAGGTTGTGCTTTT-3’  5’-TCACCTTCAATCGACACAGA-3’ |
| *ΔrrsRLK_hy_* -F  *ΔrrsRLK_hy_* -R | 5’-ATAGTCTATTCCAGTTACCTTCGAT-3’  5’-AAATATTTTCCTGTGTTGATGTGA-3’ |
| *OsPEX11* -F  *OsPEX11* -R | 5’-TCTACTGCTGGATGGCCTCT-3’  5’-AGCATGTTGGGGAGTAGTGG-3’ |

^a^ F, forward primer; R, reverse primer.
