# Supplementary material for: Lack of a Cytoplasmic RLK, Required for ROS Homeostasis, Induces Strong Resistance to Bacterial Leaf Blight in Rice
Source: Front Plant Sci. 2018 May 18;9:577. doi: 10.3389/fpls.2018.00577 (PMC5968223; doi:10.3389/fpls.2018.00577)
Supplement: Supplementary file 3 [file Table_3.DOCX]

Table S3 List of primers for cloning

| **Gene** | **Primer sequence** |
| --- | --- |
| *ΔrrsRLK* (CO-IP) -F  *ΔrrsRLK* (CO-IP) -R | 5’-TTGTCGACTACTTCCAGCCTCA-3’  5’-AAGGATCCATTCCTTGGGCTATTCCTAA-3’ |
| *OsVOZ1* (CO-IP) -F  *OsVOZ1* (CO-IP) -R | 5’-GTTAACATGTTCTCCGGGCT-3’  5’-AAATATTTTCCTGTGTTGATGTGA-3’ |
| *OsPEX11* (CO-IP) -F  *OsPEX11* (CO-IP) -R | 5’-GTTAACATGAGCACGTTAGATGCC-3’  5’-GGGGTACCTGCCTTCACTTTGATAGC-3’ |
| *ΔrrsRLK* (Y2H) -F  *ΔrrsRLK* (Y2H) -R | 5’-CACCCTTAAAACCAAGAATGAC-3’  5’-TCAATTCCTTGGGCTATT-3’ |
| *ΔrrsRLK* (Local) -F  *ΔrrsRLK* (Local) -R | 5’-GGCCCGGGGTACTTCCAGCCTCA-3’  5’-CCACTAGTGCTTAGAGATGAGA-3’ |
| *OsVOZ1* (Local) -F  *OsVOZ1* (Local) -R | 5’-TCCCCCGGGATGTTCTCCGGGCT-3’  5’-TCCCCCGGGATGGCCGGCGATCC-3’ |

^a^ Co-Immunoprecipitation (CO-IP), Yeast two hybrid (Y2H), Localization (Local)
