# Supplementary material for: Lack of a Cytoplasmic RLK, Required for ROS Homeostasis, Induces Strong Resistance to Bacterial Leaf Blight in Rice
Source: Front Plant Sci. 2018 May 18;9:577. doi: 10.3389/fpls.2018.00577 (PMC5968223; doi:10.3389/fpls.2018.00577)
Supplement: Supplementary file 4 [file Table_4.DOCX]

Table S4 List of primers for RT-PCR and qRT-PCR analysis

| **PR genes** | |
| --- | --- |
| **Gene** | **Primer sequence** |
| *OsPR1a* -F  *OsPR1a* -R | 5’-TTATCCTGCTGCTTGCTGGT-3’  5’-GGTCGTACCACTGCTTCTCC-3’ |
| *OsPRlb* -F  *OsPRlb* -R | 5’-GATTAACTATGGAGGTATCCAAGC-3’  5’-ACGTACGCCCGTGTGTATAAATAA-3’ |
| *OsPR10a* -F  *OsPR10a* -R | 5’-AGGAATACTGCCTCTATCCAG-3’  5’-GCAGAACACATTCAGACTTGC-3’ |
| *OsPR10b* -F  *OsPR10b -R* | 5’-CACAGGTCAAACCATGTGATC-3’  5’-CTCTATCTAAGCTAGTTTCCACCTG-3’ |
| *OsPR10c* -F  *OsPR10c* -R | 5’-AGAAATGTCCAAATTCTCGT-3’  5’-ACATTCAGACTTGCCTCTCT-3’ |
| *OsWRKY67* -F  *OsWRKY67* -R | 5’-CTTCAGCAACTCCTACTCCTACT-3’  5’-TCTCGTACCTCATCATAGTGTTT-3’ |
| *OsWRKY70* -F  *OsWRKY70* -R | 5’-ACAGTACTCGGACTTCACGTT-3’  5’-GACTGCTCCACCTTCTTCTT-3’ |
| *POX22.3* -F  *POX22.3* -R | 5’-CAGGCAGCTAATCAGTAGTAG-3’  5’-ACCATGTCGGTTGCGTCGAG-3’ |
| *Osh69* -F  *Osh69* -R | 5’-CTTGTACAGTTCTAAGAGGAGTGC-3’  5’-AGATTCCATATCTTCAGGATACTCT-3’ |
| *SALT* -F  *SALT* -R | 5’-ATGACGCTGGTGAAGATTG-3’  5’-GATTGCATGAAGTACAACA-3’ |
| *OsEDS1* -F  *OsEDS1* -R | 5’-TCAGTTGGATCCCCAGCAA-3’  5’-TCCCAAGTAATCCACGCAAAC-3’ |
| *SGR* -F  *SGR* -R | 5’-CTACCAAACCGAGCCAAAAT-3’  5’-ACCAAAACGACTCTTGACAGC-3’ |
| *OsEREBP1* -F  *OsEREBP1* -R | 5’-GAGAAGAAGAAGCCCAGGT-3’  5’-AGCATCATAAGCTCTTGCAG-3’ |
| *OsLOX* -F  *OsLOX* -R | 5’-TCACCATGGAGATCAACG-3’  5’-GTCCATGTGGATGTCTGAAG-3’ |
| *OsAOS2* -F  *OsAOS2* -R | 5’-CAATACGTGTACTGGTCGAATG-3’  5’-GATATGAAGTGCTATGTACGT-3’ |
| *Lipase* -F  *Lipase* -R | 5’-TCCATTGCACTGGAGGCATC-3’  5’-TCCACCACTTGTCGAACAGC-3’ |
| *OsPAL4ZB8* -F  *OsPAL4ZB8* -R | 5’-AGCACATCTTGGAGGGAAGCT-3’  5’-GCGCGGATAACCTCAATTTG-3’ |
| *OsPAD4* -F  *OsPAD4* -R | 5’-TGTTGAGCCACTTGACATTG-3’  5’-AACTTGTGCAAGTCCTCCAG-3’ |
| *RBBI4* -F  *RBBI4* -R | 5’-GAGAACTAGCTAGCTACAGA-3’  5’-CAAGCGAACAGGGACAAAT-3’ |

| **Neighbor genes of *rrsRLK*** | | |
| --- | --- | --- |
| **Gene** | **Primer sequence** | |
| *ΔrrsRLK* (N1) -F  *ΔrrsRLK* (N1) -R | | 5’-GTACTTGCTCGTGGTGGACA-3’  5’-TGCAGCCTTACTAAAGTCGTGT-3’ |
| *ΔrrsRLK* (N2) -F  *ΔrrsRLK* (N2) -R | | 5’-AGTCATCAAAGCCTGTGCGA-3’  5’-GACCATCGCCAGGAAGGAAA-3’ |
| *ΔrrsRLK* (N3) -F  *ΔrrsRLK* (N3) -R | | 5’-TTGGCAAAACGAGCCAAACA-3’  5’- ACAGAACCGTAACCACCCTG -3’ |

^a^ N1: Leucine rich repeat family protein, LOC_Os01g02280, N2: Retrotransposon protein,

LOC_Os01g02230, and N3: Protein kinase domain containing protein, LOC_Os01g02320

| **Hormone related genes** | | |
| --- | --- | --- |
| **Gene** | **Primer sequence** | |
| *OsPAL1* -F  *OsPAL1* -R | | 5’-ATCCTCGCCGGGAGCTCGTT-3’  5’-CCTGGCCGAGACGAGACCCA-3’ |
| *OsICS1* -F  *OsICS1* -R | | 5’-TGTTGAGCCACTTGACATTG-3’  5’-AACTTGTGCAAGTCCTCCAG-3’ |
| *OsAOS2* -F  *OsAOS2* -R | | 5’-AAGGACGCCTTCGTCCCGGT-3’  5’-CACCGTCGCGAACAGCAGGT-3’ |
| *OsJMT1* -F  *OsJMT1* -R | | 5’-TGCTCGCAACTCCACAGTTCAGAAA-3’  5’-TCGCCAAGCTTTTCGCGACG-3’ |
| *OsACO7* -F  *OsACO7* -R | | 5’-GGCGTGGAGGCGGCGTTAAT-3’  5’-GCCGAACTTGGTGCCCTGGT-3’ |
| *OsACS1* -F  *OsACS1* -R | | 5’-AGCCGCAGCTGCTGTCCAAG-3’  5’-AAAGCCGGGAGGCCGTGGT-3’ |

| ***rrsRLK*, *OsVOZ1*, and *OsPEX11* Expression test** | | |
| --- | --- | --- |
| **Gene** | **Primer sequence** | |
| *ΔrrsRLK* -F  *ΔrrsRLK* -R | | 5’-GGTGGCCTCTGCACTCTATC-3’  5’-TCCTAATGCTTGTTCCTTCGGA-3’ |
| *OsVOZ1* -F  *OsVOZ1* -R | | 5’-CGCGGTGTTCCATGATCAGATG-3’  5’-GGTGGACCCTCTTCCTTCA-3’ |
| *OsPEX11* -F  *OsPEX11* -R | | 5’-CCTCTTCAGTCTGTGCAGGG-3’  5’-AATGCCCCTGTTACCCTTGG-3’ |
