# Supplementary material for: Lack of a Cytoplasmic RLK, Required for ROS Homeostasis, Induces Strong Resistance to Bacterial Leaf Blight in Rice
Source: Front Plant Sci. 2018 May 18;9:577. doi: 10.3389/fpls.2018.00577 (PMC5968223; doi:10.3389/fpls.2018.00577)
Supplement: Supplementary file 5 [file Data_Sheet_1.docx]

**Supplementary materials**

**Lack of a cytoplasmic RLK, required for ROS homeostasis, induces strong resistance to bacterial leaf blight in rice**

Youngchul Yoo^1, ✝^, Jong-Chan Park^1, ✝^, Man-Ho Cho^1^, Jungil Yang  ^1, 2^, Chi-Yeol Kim^1, 3^, Ki-Hong Jung^1^, Jong-Seong Jeon^1^, Gynheung An^1^, and Sang-Won Lee^1 *^

This file includes:

Supplementary methods

- Pathogen inoculation and disease evaluation
- Genotyping of mutant plants
- Thermo cycling conditions for RT-PCR and real-time PCR
- Generation of a recombinant protein including a tyrosine kinase domain of rrsRLK and tyrosine kinase activity assay
- Isolation and transformation of protoplast from rice mesophyll and O.C suspension cells
- Activity assay of trypsin inhibitor
- In situ detection of hydrogen peroxide (H_2_O_2_)
- Specific activity of the antioxidant enzymes
- Preliminary test for expression of the neighbor genes of *Os1g02290*

References

Figures S1 to S8

**Supplementary methods**

**Pathogen inoculation and disease evaluation**

*Xoo* strains were cultured for three days on PSA plates containing cephalexin (15 μg/L) and were suspended to 10^9^ CFU (colony forming unit)/mL in water for inoculation using the clipping method (Kauffman *et al*., 1973). Generally, three healthy, recently and fully developed leaves from each tiller of individuals were chosen for inoculation. To evaluate the resistance of each rice line against the *Xoo* strains, lesion lengths were scored 14 days after inoculation (DAI), and the bacterial population in the inoculated leaves was measured after extraction by colony counting. To collect the bacteria cells from the inoculated leaves, the leaf was detached from the rice, cut into thin slices, suspended, and shaken for 30 min in 10 mL of water containing cephalexin (15 mg/mL). After serial dilutions, the supernatant was dropped onto the PSA plate, the plate was cultured at 28°C, and the colonies were counted within three days.

*M.* *grisea* inoculation was carried out using the leaf punch inoculation method (Lee *et al*., 2009). Conidia were induced for five days by scratching the plate surface with a sterilized loop and placing agar blocks covered with spores on injured spots of 2.0 mm diameter, three-week-old rice leaves. The inoculated rice leaves were placed in dark, sealed containers to maintain humidity at 24°C for 24 hr and then incubated at the same relative humidity under 14/10 hr (light/dark) photoperiods. For disease evaluation, blast lesion lengths on the leaves were scored at 10 DAI.

**Genotyping of mutant plants**

T-DNA homozygous insertion was investigated by PCR in T_1_ and T_2_ populations of each mutant line. PCR genotyping was used to determine segregation families and was carried out using specific primer sets for each gene of each mutant line. Rice leaves were pulverized in liquid nitrogen and suspended in an extraction buffer (100 mM Tris-HCl, pH 8.0, 1.4 M NaCl, 20 mM EDTA, 2% CTAB, 2% PVP40, 0.2% β-mercaptoethanol). After successive extraction with phenol/chloroform (1:1, v/v), the aqueous phase was concentrated by ethanol precipitation. The pellet was then washed in 70% ethanol and re-suspended in TE buffer (10 mM Tris-HCl, pH 7.4, 1 mM EDTA). The PCR reaction was carried out in a 20 μL of mixture containing 20 ng of the genomic DNA extracted from each line, 2 μL of 10X PCR buffer (Takara, Japan), 250 μM dNTP, 0.5 units of ExTaq polymerase (Takara, Japan), and 1 µM of the specific primers for each gene. The PCR conditions were set at 94°C for 5 min as an initial step, followed by 35 cycles of 94°C for 1 min, 58°C for 30 sec, and 72°C for 1 min. All primers for genotyping the mutant lines used in this study are listed in Table S2.

**Thermo cycling conditions for RT-PCR and real-time PCR**

cDNA was synthesized with 2 μg of the extracted RNA using the cDNA EcoDry^TM^ premix (Clontech, USA). RT-PCR was carried out in 20 μL of reaction mixture containing 100 ng of the synthesized cDNA, 1 unit of Hotstart DNA polymerase (Bioneer, Korea), 2 μL of 10X PCR buffer, 250 μM of dNTP (Bioneer, Korea), and 0.1 μM primers for each target gene. The PCR program was initiated with the denaturation step at 94°C for 5 min, followed by 25 cycles at 94°C for 30 sec, 58°C ± X (differences depended on the target genes) for 30 sec, 72°C for 30 sec, and 72°C for 30 sec for final polymerization. All of the PCR reactions in this study used *Actin1* and/or *Ubiqutin1* (Jain e*t al*., 2006) as internal controls. RT-PCR was repeated more than three times with three biological replicates.

All real-time PCR reactions were carried out using the rotor-gene Q system (Qiagen, Germany), and all amplifications were done using the SYBR Premix Ex Taq (TaKaRa, Japan). The thermal cycling conditions were followed by an initial denaturation step at 94°C for 5 min, 45 cycles at 94°C for 30 sec, 58°C ± X (differences depended on target genes) for 30 sec, and 72°C for 30 sec. Using the 2^-∆∆Ct^ method (Livak *et al*., 2001), we obtained the fold changes in gene expression normalized to an internal control gene.

**Generation of a recombinant protein including a tyrosine kinase domain of rrsRLK and tyrosine kinase activity assay**

The tyrosine kinase domain (aa 325 -393) of rrsRLK protein was expressed in *E. coli* (rrsRLK_k_). The plasmid containing a fragment of *rrsRLK* (nt 859 to 1197) encoding a kinase domain was amplified by PCR using Pfu polymerase and inserted into pET28-a vector (Novagen, Germany) after digestion with *BamH*I and *Sac*I. The primers for the PCR were 5’-GGATCCCTTAAAACCAAGAATGAC-3’ containing a *BamH*I recognition sequence at the 5’ end and 5’-GAGCTCTCAATTCCTTGGGCTATT-3’ containing a *SacI* recognition sequence at the 5’ end. The complete construct was introduced into *E. Coli* BL21 (DE3), and the transformant was cultured in 10 mL of Luria-Bertani (LB) medium containing kanamycin (50 μg/mL) at 37°C overnight. The seed culture was transferred into 1 L of LB containing kanamycin (50 μg/mL) and incubated until the OD_600_ reached 0.6. Expression of the recombinant protein were induced by adding isopropyl β-D-1-thiogalactopyranoside (IPTG) to a final concentration of 1 mM, and the cells were further cultivated for 4 hours. The cells were harvested with centrifugation and re-suspended in 1 mL 50 mM NaH_2_PO_4_ (pH 8.0) containing 500 mM NaCl. After sonication, the lysate was clarified by centrifugation (12000 rpm for 15 min at 4°C) and loaded onto a Ni-NTA agarose column equilibrated with the lysis buffer. The column was washed five times with washing buffer (50 mM NaH_2_PO_4_, pH 8.0, 500 mM NaCl, 150 mM imidazole). The protein was eluted with elution buffer (50 mM NaH_2_PO_4_, pH 8.0, 500 mM NaCl, 250 mM imidazole). The purification of rrsRLK_k_ was confirmed by SDS-PAGE and Western blot. Tyrosine kinase activity assay was carried out with a universal tyrosine kinase assay kit (TaKaRa, japan) according to the manufacturer`s instructions. Briefly, tyrosine kinase substrate immobilized on a micro-plate and kinase reacting solution containing 10 mM 2-mercaptoethanol were added. Ten microliters of 40 mM ATP-2Na solution was further added and incubated for 30 min at 37°C, immediately after addition of 2 μg of the purified rrsRLK_k_. The reaction was terminated with 4 washes and additions of 100 μL blocking solution. Anti-phosphotyrosine (PY20)-HRP solution (50 μL) was added to the wells, and the plate was incubated for 30 min at 37°C, washed 4 times with washing buffer, treated with 100 μL of HRP substrate solution, and then incubated for 15 min at 37°C. Absorbance at 450 nm was measured with a microplate reader (SunriseTM, TECAN, Switzerland) and tyrosine kinase activity of each sample was calculated with an equation derived from a standard curve established using a recombinant c-Src (Proto-oncogene tyrosine-protein kinase).

**Isolation and transformation of protoplast from rice mesophyll and O.C suspension cells**

The leaves were excised at 7 to 14 days after germination of rice seedlings with a 446 razor blade and incubated in enzyme solution A [1.5% (w/v) Cellulose RS (Onozuka -R-10), 0.3% (w/v) Macerozyme, 0.1% (w/v) pectolyase, 0.6 M mannitol, 10 mM MES, pH 5.7, 1 mM CaCl_2_, and 0.1% (w/v) BSA (bovine serum albumin)] for 4 hr in the dark with gentle shaking. Cells were collected from O.C suspension solution by centrifugation and incubated in enzyme solution B [2% (w/v) Cellulose RS, 1% (w/v) Macerozyme, 0.4 M mannitol, 0.1% (w/v) MES, pH 5.7, 0.1% (w/v) CaCl_2_] for 4 hr with gentle shaking. After adding an equal volume of W5 solution (154 mM NaCl, 125 mM CaC1_2_, 5 mM KCl, and 2 mM MES, pH 5.7), cells and protoplasts were re-suspended in MMG solution (0.6 M mannitol, 15 mM MgCl_2_, and 4 mM MES, pH 5.7) to 10^6^ cells/mL, as quantified with a hemocytometer. Each construct in 40% PEG solution [0.6 M mannitol, 100 mM CaCl_2_, and 40% (w/v) PEG 3350] was added to the protoplast and incubated for 10 minutes at room temperature for transformation. The samples were then incubated overnight at 28°C in the dark before microscopic examination.

**Activity assay of trypsin inhibitor**

The activity of trypsin inhibitor was measured using a previously reported method (Neuberger et al., 1987). To extract total proteins, 1 g of rice leaves was homogenized in 2 mL of 63 mM sodium phosphate buffer (pH 7.6) containing 0.5% (v/v) Triton X-100. The homogenate was filtered through four layers of gauze and centrifuged at 17,000 ×g for 25 min. The supernatant was collected for measurement of trypsin inhibitor activity, and quantitative analysis of total protein contents was carried out with the Bradford method (Lucarini et al., 1999) using bovine serum albumin (Sigma, USA) as a standard protein. Trypsin inhibitor inhibits the catalytic reaction by trypsin to produce *N*-benzoyl-L-arginine and ethanol from *N*-benzoyl-L-arginine ethyl ester. To measure the activity of the trypsin inhibitor in the protein extracts of rice, 3.2 mL of reaction mixture were prepared with a final concentration of 63 mM sodium phosphate, 0.23 mM N-benzoyl-L-arginine ethyl ester, 2 µM HCl, and 1 µg of the extracted protein. The inhibitory activity of trypsin inhibitor in rice protein extract was monitored with a spectrophotometer (A_253nm_) for 5 min immediately after addition of 5 µg trypsin. The inhibition rate of the trypsin inhibitor is defined as follows:

$$\left( A-B \right)*\frac{C}{A*D}$$

(A: *Δ*A_253nm_/minute control, B: *Δ*A_253nm_/minute sample, C: µg trypsin in reaction mixture, D: µg inhibitor in reaction mixture)

**In situ detection of hydrogen peroxide (H_2_O_2_)**

Detection of H_2_O_2_ was determined by a method that uses 3, 3ˈ- diaminobenzidine (DAB) staining (Bindshedler et al., 2006; Daudi et al., 2012; Thordal-Christensen et al., 1997). Whole plants (Dongjin, Hwayoung, *ΔrrsRLK*) were collected and filtered under gentle vacuum in 10 mM sodium phosphate buffer (pH 7.0) containing 1 mg/mL DAB and 0.05% (v/v) Tween 20. The staining reaction was terminated 6 hr after DAB infiltration, and the filtrate was fixed in bleaching solution containing ethanol/glycerol/acetic acid (3:1:1, v/v) for 20 min in a water bath at 95°C. The rice leaf, stem, and root were immersed in bleaching solution until the chlorophyll was completely bleached and observed under a BX61 microscope (Olympus, <http://www.olympus-global.com/enl/>) with variable magnifying power.

**Specific activity of the antioxidant enzymes**

The specific activity of the antioxidant enzymes was defined as follows:

$$\frac{V}{Ɛ\times d\times v\times C_{\mathrm{protein}}}\times\Delta E/\Delta t$$

V: total assay volume, Ɛ: extinction coefficient (Ɛ_290nm_ = 2.8 mM·cm^-1^, Ɛ_240nm_ = 40.0 cm^2^/μmol, Ɛ_436nm_ = 6.39 cm^2^/μmol), d: light path (1 cm), v: volume of sample used in the assay, C _protein_: concentration of protein (μg/mL).

**References**

Bindschedler, L.V., Dewdney, J., Blee, K.A., Stone, J.M., Asai, T., Plotnikov, J., et al. (2006). Peroxidase‐dependent apoplastic oxidative burst in Arabidopsis required for pathogen resistance. *Plant J*. 47(6), 851-863.

Daudi, A., and O’Brien, J.A. (2012). Detection of hydrogen peroxide by DAB staining in Arabidopsis leaves. *Bio*-*protocol* 2(18).

Jain, M., Kaur, N., Garg, R., Thakur, J.K., Tyagi, A.K., and Khurana, J.P. (2006). Structure and expression analysis of early auxin-responsive Aux/IAA gene family in rice (Oryza sativa). *Funct*. *Integr*. *genomic* 6(1), 47-59.

KauffmanHE, R. (1973). An improved technique for evaluating resistance of rice varieties to *Xanthomonas* *oryzae*. *Plant Dis. Rep.* 5(7)**,** 5.

Lee, S.-K., Song, M.-Y., Seo, Y.-S., Kim, H.-K., Ko, S., Cao, P.-J., et al. (2009). Rice Pi5-mediated resistance to Magnaporthe oryzae requires the presence of two coiled-coil–nucleotide-binding–leucine-rich repeat genes. *Genetics* 181(4), 1627-1638.

Livak, K.J., and Schmittgen, T.D. (2001). Analysis of relative gene expression data using real-time quantitative PCR and the 2− ΔΔCT method. *methods* 25(4), 402-408.

Lucarini, A., and Kilikian, B. (1999). Comparative study of Lowry and Bradford methods: interfering substances. *Biotechnol*. *Tech*. 13(2), 149-154.

Neuberger, A., and Brocklehurst, K. (1987). Hydrolytic enzymes. *Elsevier*.

Thordal-Christensen, H., Zhang, Z., Wei, Y., and Collinge, D.B. (1997). Subcellular localization of H2O2 in plants. H2O2 accumulation in papillae and hypersensitive response during the barley—powdery mildew interaction. *Plant J.* 11(6), 1187-1194.

**Preliminary test for expression of the neighbor genes of the *Os1g02290***

To test if the resistance in the selected mutants *ΔrrsRLK_dj_* and *ΔrrsRLK_hy_* against *Xoo* was not induced by suppression of the *Os1g02290* gene, but from expressional changes of the neighbor genes (Leucine rich repeat family protein, LOC_Os01g02280; Retrotransposon protein, LOC_Os01g02230; Protein kinase domain containing protein, LOC_Os01g02320), RT-PCR experiments were carried out with specific primers for each gene and RNA extracted from wild type and *ΔrrsRLK_dj_*. As shown below, no changes in expression of the genes were observed, indicating that the T-DNA insertion in *ΔrrsRLK_dj_* did not induce expression of the neighbor genes.


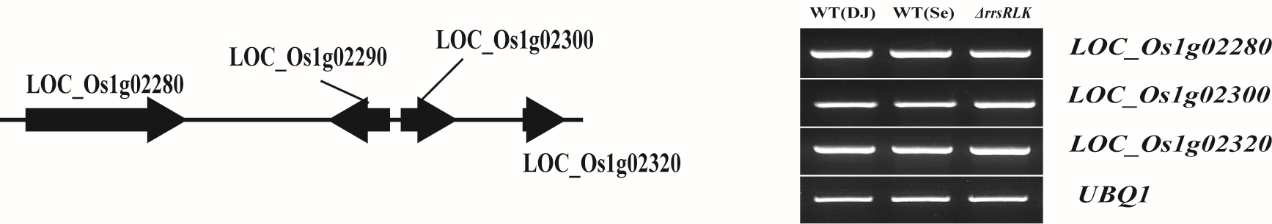


**Supplementary Figure legends**

**
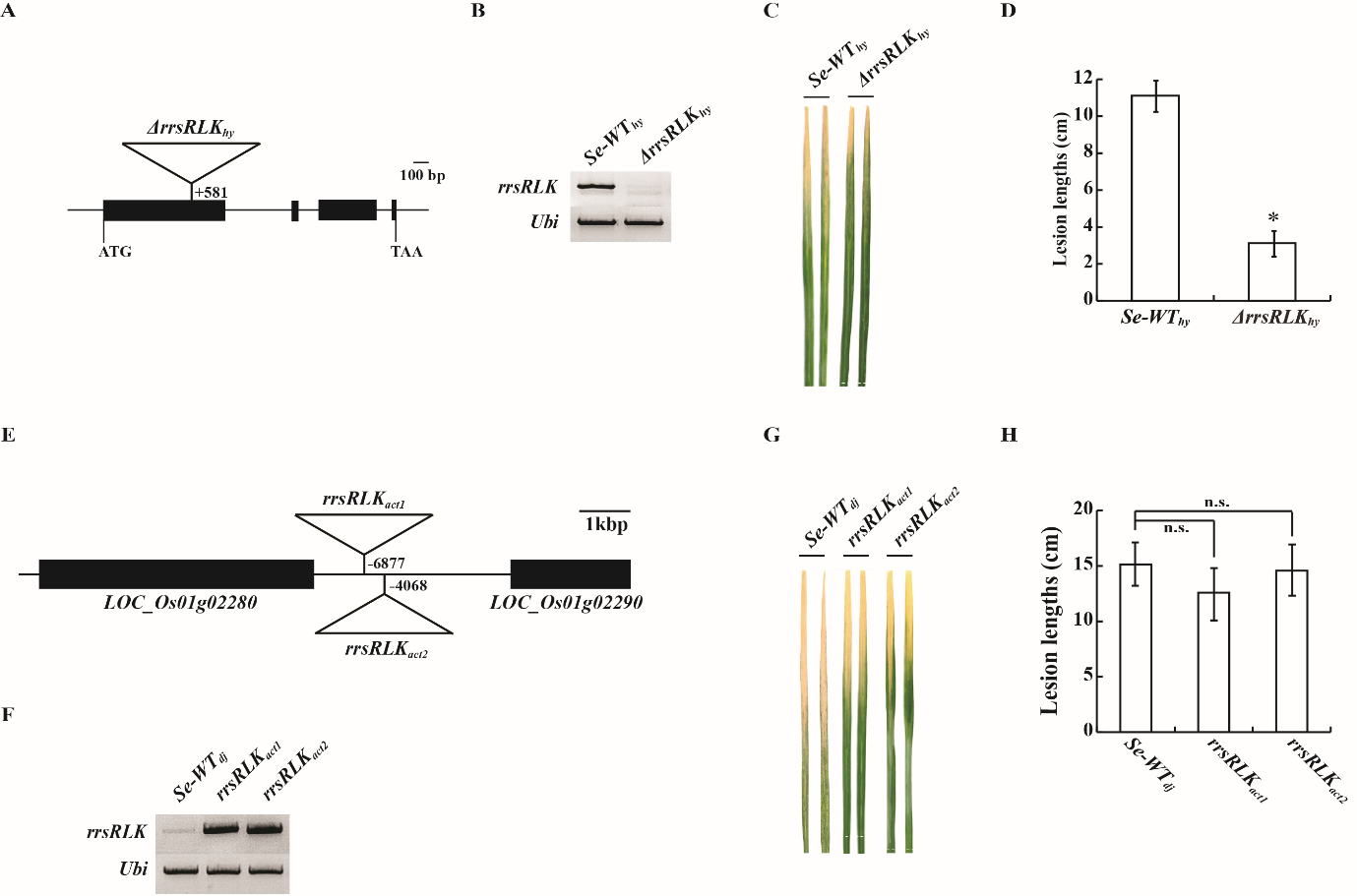
Figure S1**

**Figure S1.** (A) Schematic representation of the T-DNA insertion (+581 bp from the translational start site) in *Os01g02290* encoding rrsRLK in Hwayoung (*ΔrrsRLK_hy_*). ATG start codon and TGA stop codon are indicated. *Os01g02290* consists of four exons (black boxes) and three introns (line between the black boxes). The black bar indicates 100bp. (B) Expression analysis of the *rrsRLK* gene in *Se-WT_hy_* (segregated wild type) and *ΔrrsRLK_hy_* mutant lines using RT-PCR with *rrsRLK* primers. *Ubi* gene was used as an internal control. The experiment was repeated three times with consistent results. (C) Comparison of lesion development on leaves of *Se-WT_hy_* and homozygous mutant lines of *ΔrrsRLK_hy_*. Rice plants grown for six weeks were inoculated with HB01009 (K3a), and lesion development was monitored for 14 DAI. The results were consistent until the 6^th^ generation. Each experiment was carried out with more than 40 leaves. (D) Lesion lengths scored at 14 DAI on leaves of *Se-WT_hy_* and *ΔrrsRLK_hy_* mutant lines. The averages and error ranges of each sample were calculated from 40 leaves. The asterisk indicates P < 0.05 (Student’s t-test). Data in (C) and (D) are results carried out with 3^rd^ generation *ΔrrsRLK_hy_* mutant lines. (E) Schematic representation of the T-DNA insertion (*ΔrrsRLK_act1_*: -6877bp from the translational start site and *ΔrrsRLK_act2_*: -4068bp from the translational start site) at *Os01g02290* encoding rrsRLK in Dongjin (*ΔrrsRLK_act1_* and *ΔrrsRLK_act2_*). The figure consists of two black box genes (*LOC_Os01g02280* and *LOC_Os01g02290*). The black bar indicates 1 kbp. (G) Comparison of lesion development on leaves of *Se-WT_dj_* and two activation mutant lines (*ΔrrsRLK_act1_*, *ΔrrsRLK_act1_*). Rice plants grown for six weeks were inoculated with PXO99az, and lesion development was monitored for 14 DAI. The results were consistent until the 6^th^ generation of the mutant. Each experiment was carried out with more than 40 leaves. (H) Lesion lengths scored at 14 DAI on leaves of *Se-WT_dj_*, *ΔrrsRLK_act1_*, and *ΔrrsRLK_act2_* mutant lines. The averages and error ranges of each sample were calculated from 40 leaves. The asterisk indicates P < 0.05 (Student’s t-test). Data in (G) and (H) are results carried out with 3^rd^ generation of *ΔrrsRLK_dj_* mutant lines. (F) Expression analysis of *rrsRLK* gene in *Se-WT_dj_* (segregated wild type), *ΔrrsRLK_act1_*, and *ΔrrsRLK_act2_* mutant lines using RT-PCR with *rrsRLK* primers. *Ubi* gene was used as an internal control. The experiment was repeated three times with consistent results.

**Figure S2**

**
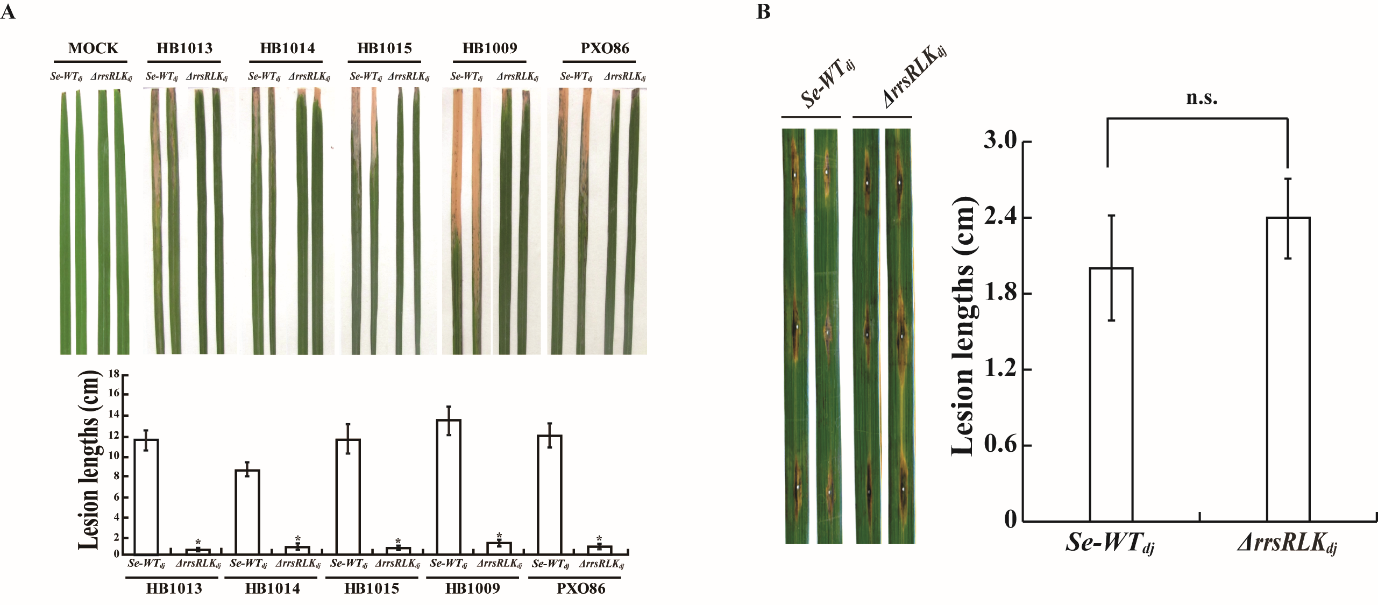
**

**Figure S2.** (A) Inoculation experiment and lesion lengths on leaves of *ΔrrsRLK_dj_* and *Se-WT_dj_*. Rice plants were grown for six weeks and inoculated by *Xoo* strains including Korean strains (HB01009, HB01013, HB01014, HB01015) and a Philippine strain, PXO86. Lesion development on the leaves was monitored (up) and lengths were scored at 14 DAI (down). This experiment was repeated three times with more than 15 leaves for each and the results were consistent until the 6^th^ generation of the mutant. The asterisk indicates P < 0.05 (Student’s t-test). This result is the representative one of three repeats carried with 3^rd^ generation *ΔrrsRLK_dj_* mutant lines and the leaves inoculated with water (MOCK) was used as a control. (B) Comparison of lesion development on leaves of *Se-WT_dj_* and homozygous mutant lines of *ΔrrsRLK_dj_*. Rice plants grown for three weeks were inoculated with *Magnaporthe grisea* (*PO6-6*) and monitored for lesion development for 10 DAI. Lesion lengths scored at 10 DAI on leaves of *Se-WT_dj_* and *ΔrrsRLK_dj_* mutant lines. The results were consistent until the 6^th^ generation of the mutant. The averages and error ranges of each sample were calculated from 40 leaves. The asterisk indicates P < 0.05 (Student’s t-test). The results of 3^rd^ generation *ΔrrsRLK_dj_* mutant lines.

**Figure S3**

**
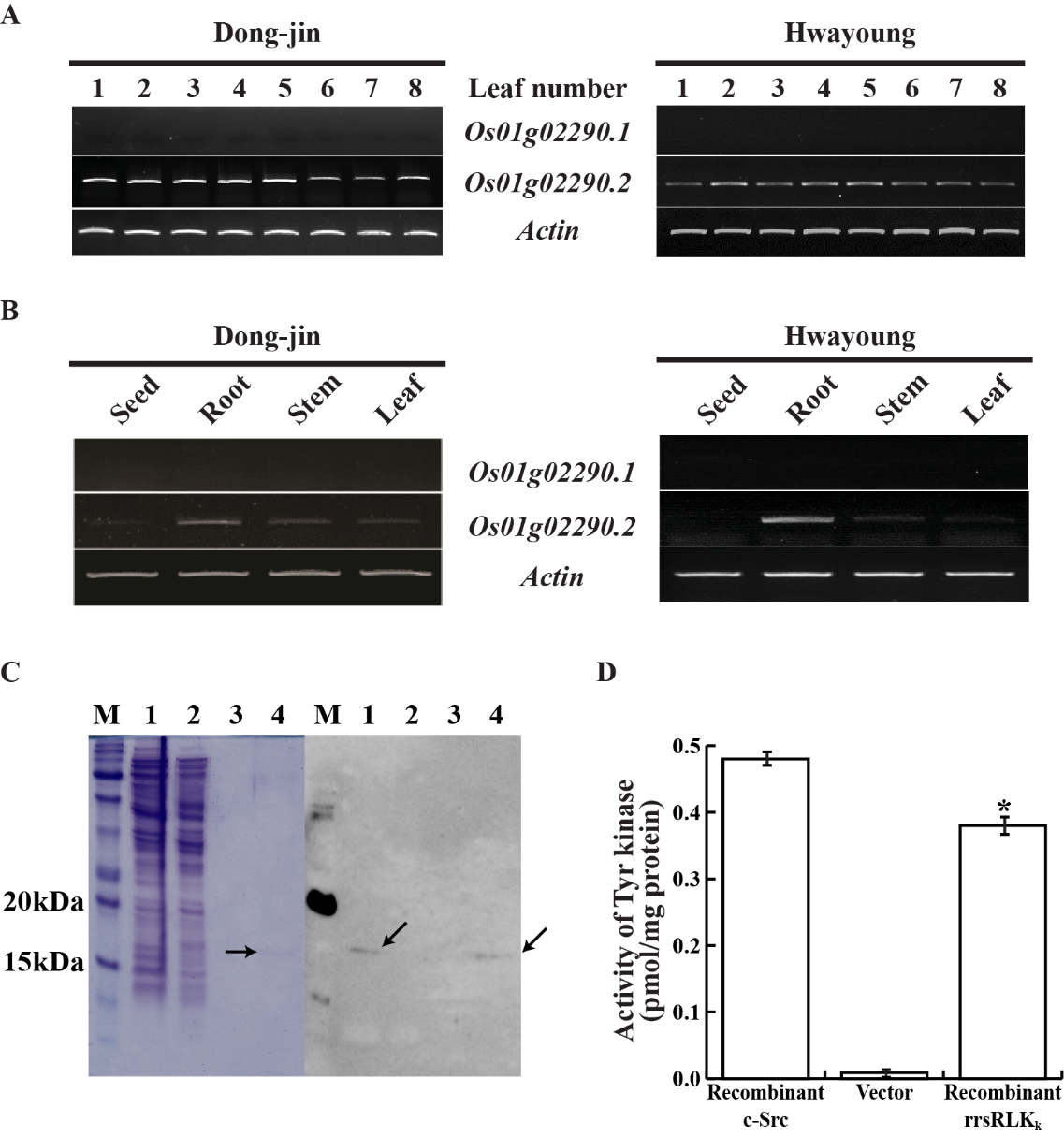
**

**Figure S3.** (A) Expression analysis of alternative splicing *rrsRLK* genes (*Os01g02290.1* and *Os01g02290.2*) in the developmental stage of Dongjin and Hwayoung using RT-PCR with *rrsRLK* gene primers. The experiment was repeated three times with consistent results. Samples were harvested at various developmental stages (1 leaf to 8 leaf stages) from greenhouse conditions and used for RNA extraction. *Actin* gene was used as an internal control. (B) Comparison of *Os01g02290.1* and *Os01g02290.2* gene expression in various tissues. Dongjin and Hwayoung samples were used. The experiment was repeated three times with consistent results. (C) Purification of recombinant rrsRLK in coomassie blue staining and western blot. 1: Lysate supernatant, 2: pET28a vector, 3: pET-28a purification, 4: Recombinant rrsRLK-kinase (rrsRLK_k_). The black arrow indicates the expressed recombinant rrsRLK_k_. (D) Verification of tyrosine kinase activity of rrsRLK_k_. Lane 1: Recombinant c-Src (Proto-oncogene tyrosine-protein kinase), lane 2: empty vector (pET28-a), lane 3: purified recombinant rrsRLK_k_. Concentration of the recombinant rrsRLK_k_ protein was determined according to the Bradford method using bovine serum albumin as a standard protein. Different letters above bars indicate statistically significant differences as determined by one-way analysis of variance (ANOVA), P < 0.05 (*).

**Figure S4**

**
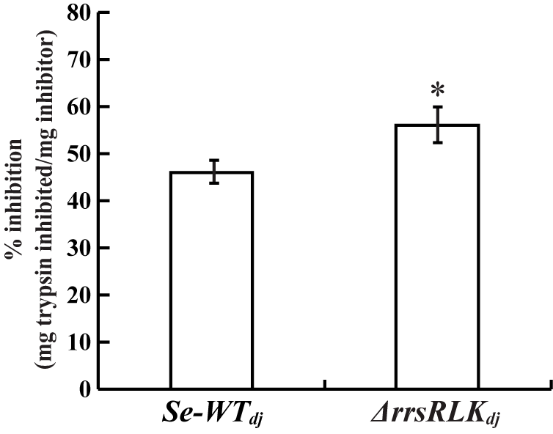
**

**Figure S4.** Enzymatic assay of trypsin-inhibition activity in leaves of *Se-WT_dj_* and *ΔrrsRLK_dj_* leaves. For this assay, 1 µg total protein in each sample was used. The concentration of total protein in extract was determined according to the Bradford method using bovine serum albumin as a standard protein. The experiment was repeated three times with consistent results. Different letters above bars indicate statistically significant differences as determined by one-way analysis of variance (ANOVA), P < 0.05, Values are mean ± SD (n=9).

**Figure S5**

**
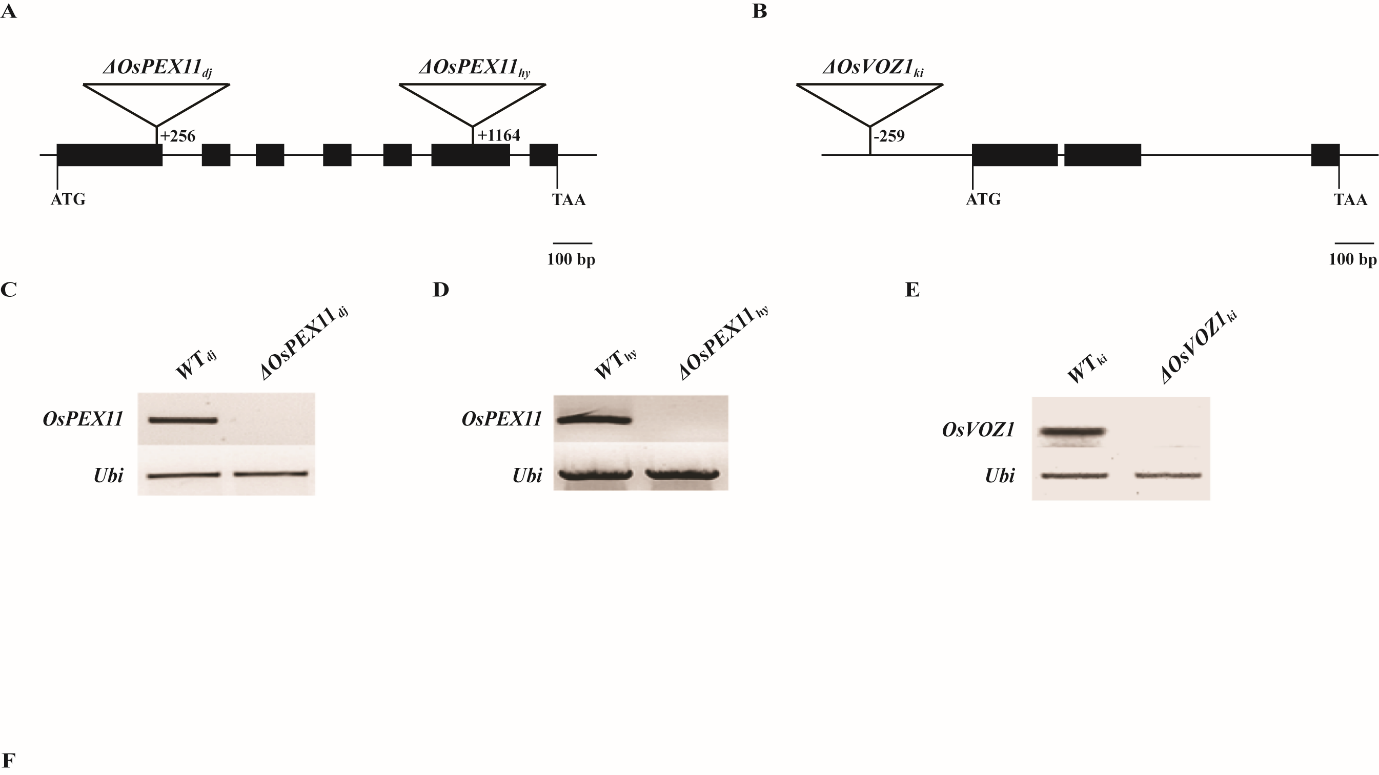
**

**Figure S5.** (A) Schematic representation of the T-DNA insertion (*ΔOsPEX11_dj_*: +256 bp from the translational start site and *ΔOsPEX11_hy_*: +1164 bp from the translational start site) at *Os03g02590* encoding PEX11 in Dongjin and Hwayoung (*ΔOsPEX11_dj_* and *ΔOsPEX11_hy_*). ATG start codon and TGA stop codon are indicated. *Os03g02590* consists of seven exons (black boxes) and six introns (line between the black boxes). The black bar indicates 100bp. (B) Schematic representation of the T-DNA insertion (*ΔOsVOZ_ki_*: -259 bp from the translational start site) at *Os01g54930* encoding VOZ1 in Kittake. ATG start codon and TGA stop codon are indicated. *Os01g54930* consists of three exons (black boxes) and two introns (line between the black boxes). The black bar indicates 100bp. The expression analysis of *ΔOsPEX11* (C, D) and *ΔOsVOZ* (E) genes at three weeks in *WT_dj_*, *ΔOsPEX11_dj_*, *ΔOsPEX11_hy_*, and *ΔOsVOZ* _ki_ plants. *Ubi* gene was used as an internal control. The experiment was repeated three times with consistent results.

**Figure S6**

**
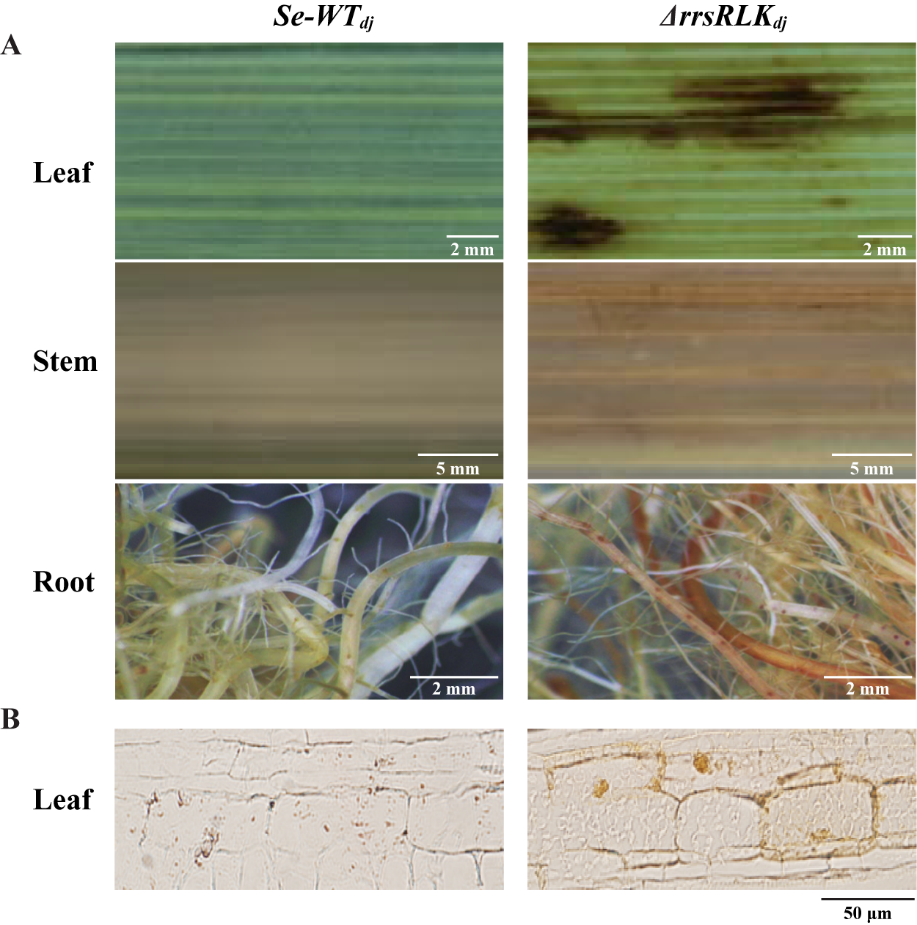
**

**Figure S6.** *In vivo* DAB staining. (A) Tissue polymerization of DAB is confirmed by a dark staining (reddish-brown) reaction in the leaves, stems, and roots of *Se-WT_dj_* and *ΔrrsRLK_dj_* rice plants. The staining reaction was terminated 6 h after DAB infiltration, and the tissues were then fixed in bleaching solution containing ethanol/glycerol/acetic acid (3:1:1, v/v) for 20 min in a water bath at 95°C. The rice samples were immersed in bleaching solution again until the chlorophyll was completely bleached. The white bars indicate 2 mm and 5 mm. (B) Cross-sections of leaf blades. The stained samples were observed with a dissecting BX61 microscope (Olympus, http://www.olympus-global.com/enl/). For longitudinal and transverse sectioning, the stained samples were embedded in Paraplast Plus (Sigma), and 10 µm sections were prepared using a microtome (Microm HM360). The sections mounted on slides were observed with a compound microscope. The black bar indicates 50 µm.

**Figure S7**

**
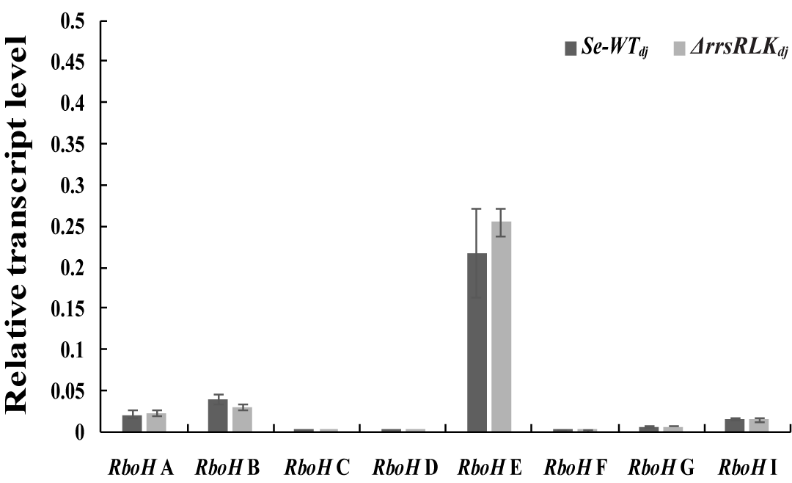
**

**Figure S7.** Relative transcript level of rice *RBOH* genes (A, B, C, D, E, F, G, and I) in leaf tissue. Real-time qRT-PCR analysis was carried out to test expression of rice *RBOH* genes at leaves of *Se-WT_dj_* and *ΔrrsRLK_dj_* rice plants. Expression of each gene was normalized to that of *Ubi* gene and compared with that of *Se-WT_dj_* rice plants. Values represent mean ± standard deviation of three repetitions of three biological replicates (n = 9).

**Figure S8**

**
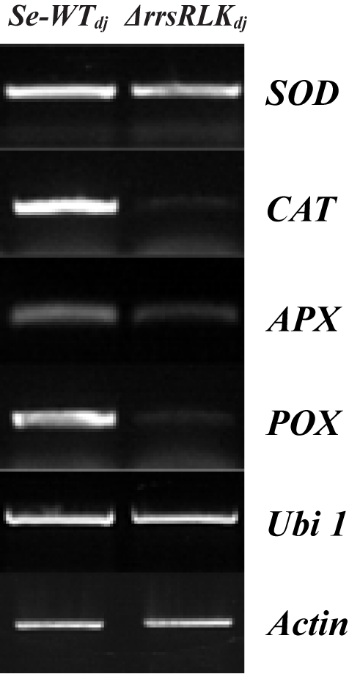
**

**Figure S8.** Expression of genes encoding ROS-scavenging enzymes in *Se-WT_dj_* and *ΔrrsRLK_dj_* plants without *Xoo* infection. The plants were grown in a greenhouse for 3 weeks. Transcript level of four genes (*SOD*, *CAT*, *APX,* and *POX*) were determined by RT-PCR. The rice *Ubiquitin* (*Ubi1*) and *Actin* genes were used as internal controls. This picture is a representative of three repeats that showed consistent results.
